# Aspf2 From *Aspergillus fumigatus* Recruits Human Immune Regulators for Immune Evasion and Cell Damage

**DOI:** 10.3389/fimmu.2018.01635

**Published:** 2018-08-03

**Authors:** Prasad Dasari, Iordana A. Shopova, Maria Stroe, Dirk Wartenberg, Hans Martin-Dahse, Niklas Beyersdorf, Peter Hortschansky, Stefanie Dietrich, Zoltán Cseresnyés, Marc Thilo Figge, Martin Westermann, Christine Skerka, Axel A. Brakhage, Peter F. Zipfel

**Affiliations:** ^1^Department of Infection Biology, Leibniz Institute for Natural Product Research and Infection Biology, Hans Knöll Institute (HKI), Jena, Germany; ^2^Department of Molecular and Applied Microbiology, Leibniz Institute for Natural Product Research and Infection Biology (HKI), Jena, Germany; ^3^University of Würzburg, Institute for Virology and Immunobiology, Würzburg, Germany; ^4^Research Group Applied Systems Biology, Leibniz Institute for Natural Product Research and Infection Biology (HKI), Jena, Germany; ^5^Faculty for Biological Sciences, Friedrich Schiller University, Jena, Germany; ^6^Electron Microscopy Center of the University Hospital, Jena, Germany

**Keywords:** complement, blocking opsonization, phagocytosis, acquisition of host regulators, immune evasion

## Abstract

The opportunistic fungal pathogen *Aspergillus fumigatus* can cause life-threatening infections, particularly in immunocompromised patients. Most pathogenic microbes control host innate immune responses at the earliest time, already before infiltrating host immune cells arrive at the site of infection. Here, we identify Aspf2 as the first *A. fumigatus* Factor H-binding protein. Aspf2 recruits several human plasma regulators, Factor H, factor-H-like protein 1 (FHL-1), FHR1, and plasminogen. Factor H contacts Aspf2 *via* two regions located in SCRs6–7 and SCR20. FHL-1 binds *via* SCRs6–7, and FHR1 *via* SCRs3–5. Factor H and FHL-1 attached to Aspf2-maintained cofactor activity and assisted in C3b inactivation. A Δ*aspf2* knockout strain was generated which bound Factor H with 28% and FHL-1 with 42% lower intensity. In agreement with less immune regulator acquisition, when challenged with complement-active normal human serum, Δ*aspf2* conidia had substantially more C3b (>57%) deposited on their surface. Consequently, Δ*aspf2* conidia were more efficiently phagocytosed (>20%) and killed (44%) by human neutrophils as wild-type conidia. Furthermore, Aspf2 recruited human plasminogen and, when activated by tissue-type plasminogen activator, newly generated plasmin cleaved the chromogenic substrate S2251 and degraded fibrinogen. Furthermore, plasmin attached to conidia damaged human lung epithelial cells, induced cell retraction, and caused matrix exposure. Thus, Aspf2 is a central immune evasion protein and plasminogen ligand of *A. fumigatus*. By blocking host innate immune attack and by disrupting human lung epithelial cell layers, Aspf2 assists in early steps of fungal infection and likely allows tissue penetration.

## Introduction

*Aspergillus* species, as ubiquitous saprophytes, are optimally adjusted to living in a variety of environmental niches. *Aspergillus fumigatus* is an opportunistic fungal pathogen that can cause a broad spectrum of diseases, such as hypersensitivity pneumonitis, allergic asthma (1), and allergic bronchopulmonary aspergillosis (ABPA), particularly in immune-compromised individuals. *A. fumigatus* can also cause invasive aspergillosis, which has a high rate of morbidity and mortality (2).

Immune crosstalk between *A. fumigatus* and the host immediately starts upon infection, when the pathogen reaches the lung tissue. This crosstalk includes control of innate immune attack by the host, blockade of the complement system, and damage of epithelial cells that line up the lung tissue at sites of infection. *Aspergillus* conidia and hyphae activate all three pathways of the complement system (3), and resting conidia predominantly activate the alternative pathway (AP) (3–5). Mannose-binding lectin (MBL), ficolins, and pentraxin 3 are important host factors for fungal recognition and clearance (6, 7). Individuals with polymorphisms in MBL or pentraxin 3 genes are susceptible to chronic necrotizing pulmonary aspergillosis (8, 9). Similarly, mice deficient in the complement protein C5 show increased mortality when challenged with *A. fumigatus* (10, 11).

The complement system provides the first protective defense line of innate immunity against invading microbes. It can be activated by three pathways: the alternative, the classical, and the lectin pathway (LP). The AP is initiated by binding of spontaneously generated ${\text{C}3}_{{\text{H}}_{\text{2}}\text{O}}$ to nearby molecules or surfaces (12). The classical pathway is activated by immune complexes and by C-reactive protein (13), the LP by binding of MBLs to mannans or ficolins exposed on the microbial surface (14). All three pathways converge at the C3 convertase level, and newly formed C3 convertases cleave the central complement protein C3 into C3b and C3a. Activated C3b covalently attaches to microbial surfaces and marks microbes for phagocytosis by host immune effector cells; whereas C3a displays antifungal and chemotactic activities (15, 16). C3 convertases can progress to the generation of C5 convertases, which cleaves C5 to generate C5a and C5b. C5a induces cellular inflammation, and C5b initiates the terminal complement complex (TCC), resulting in lysis of target cells (12, 17–19).

Complement activation results in opsonophagocytosis, inflammation, and/or lysis of infectious microbes. However, as uncontrolled complement action can also damage host cells and tissues, host cells utilize a series of regulators to control and prevent the attack on intact self-cells. The soluble complement regulators Factor H and Factor-H-like protein 1 (FHL-1) are the primary fluid-phase negative regulators of the AP. Factor H is a 150-kDa protein composed of 20 short conserved repeats (SCR). FHL-1, which is encoded by an alternative spliced transcript of the Factor H gene, shares with Factor H SCRs1-7 and has a unique extension of four amino acids at its *C*-terminus. Both Factor H and FHL-1 have cofactor activity for factor I-mediated inactivation of C3b and display decay acceleration activity for dissociation of the AP C3 convertase C3bBb. Thus, both Factor H and FHL-1 block inappropriate complement activation and inflammation (20).

Complement factor H-related protein 1 (FHR1) is a human plasma protein and a member of the FHR protein family. FHR1 is composed of five SCRs and forms two glycosylated versions. FHR1-β (42 kDa) has two and FHR1α (37 kDa) one carbohydrate side chain attached (20, 21). SCR1 and SCR2 of FHR1 share 34 and 42% amino acid sequence identity with SCR6 and SCR7 of Factor H, but the three C-terminal SCRs of the FHR1 show a high degree of identity. SCRs3–4 are 100% identical to SCRs18–19, and SCR5 is 98% identical to SCR 20 of Factor H (20). FHR1 inhibits C5 convertase activity and TCC formation but lacks cofactor as well as decay accelerating activity (20, 21).

Following infection, *A. fumigatus* conidia germinate in the human lung and are attacked by the host complement system. However, *A. fumigatus* has evolved evasion strategies to control host complement attack (22–24). The conidial pigment 1,8-dihydroxynaphthalene (DHN) melanin protects from C3b binding and the absence of melanin results in increased opsonophagocytosis by human neutrophils (3). Also, the fungal serine protease Alp1 cleaves and degrades the human complement proteins C3, C4, and C5, allowing the fungus to escape from complement attack (23). Furthermore, *A. fumigatus* acquires soluble human plasma regulators, including Factor H, FHL-1, C4BP (C4b-binding protein), and plasminogen and thereby limits complement deposition (22). At present no surface protein of *A. fumigatus* binding to human regulators has been identified.

Besides *A. fumigatus* and *Candida albicans*, Gram-negative, Gram-positive bacteria, *Plasmodium falciparum*, and viruses have all developed strategies to control host complement attack (25–28). Recruitment of host complement regulators by these pathogenic microbes is a common immune evasion mechanism. These diverse pathogens express specific surface proteins that recruit the central human plasma regulators Factor H, FHL-1, FHR1, C4BP, and plasminogen to their surface (26, 29). Four known complement regulator acquiring surface proteins of *C. albicans* are identified, i.e., pH-regulated antigen 1 (Pra1), glycerol-3-phosphate dehydrogenase 2, high-affinity glucose transporter 1, and phosphoglycerate mutase (Gpm1), which acquire Factor H, FHL-1, C4BP, FHR1, plasminogen, and vitronectin from human plasma (24, 30, 31). Similarly, the M protein of *Streptococcus pyogenes* and PspC of *Streptococcus pneumoniae* are multifunctional immune evasion proteins that recruit several human complement regulators and coagulation factors, such as Factor H, FHL-1, C4BP, plasminogen, fibronectin, thrombin, fibrinogen, IgA, IgG, and kininogen (32–35). The porin protein 1A of *Neisseria gonorrhoeae* (36) and CRASP1-CRASP5 proteins from *Borrelia burgdorferi* recruit Factor H, FHL-1, as well as plasminogen to their surface (37).

A wide variety of microbial pathogens express virulence factors that acquire plasminogen and attached plasminogen can be activated to plasmin, which degrades tissue components and extracellular matrices (38–40). Plasminogen is synthesized in the liver as a 92-kDa glycoprotein and circulates in plasma as a zymogen at a concentration of ca. 2 µM. Plasminogen is composed of five disulfide-bonded kringle domains, followed by a serine protease domain. The kringle domains bind to fibrin clots or cell-surface receptors. Plasminogen is converted to active plasmin by host proteases, i.e., urokinase-type plasminogen activator (uPA) and tissue-type plasminogen activator (41) and is also activated by microbial proteases such as staphylokinase of *S. aureus* (40, 42). Plasmin regulates fibrinolysis and homeostasis. Activated plasmin cleaves fibrin, dissolves blood clots, and degrades extracellular matrix components (ECM) and basal membrane proteins. Furthermore, active plasmin cleaves and inactivates human complement proteins such as C1, C3, and C5 (43). Plasminogen is acquired by many microbial pathogens, including *A. fumigatus, C. albicans, P. falciparum, B. burgdorferi, P*. aeruginosa, *S. pneumoniae*, and *S. aureus*. Active plasmin is considered to contribute to tissue invasion by degrading ECM components and by disturbing ECM integrity (22, 44–46). Recently, enolase of *A. fumigatus* was identified as a plasminogen-binding protein (47).

We are interested to identify *A. fumigatus* proteins which acquire human plasma regulators and control host complement. Given the high 43% sequence homology of Aspf2 with the multifunctional *C. albicans* immune evasion and zinc chelator Pra1 *C. albicans* Pra1, we hypothesized that *A. fumigatus* Aspf2 and *C. albicans* Pra1 share immune evasion features (48). Aspf2 is a 310 amino acid-long protein expressed on the surface of *A. fumigatus* conidia and is also secreted into the culture supernatants (48). Aspf2 binds to the ECM laminin. As one major allergen of *A. fumigatus*, Aspf2 binds IgE antibodies present in sera from ABPA and cystic fibrosis-ABPA patients (48). Furthermore, Aspf2 is a zinc acquiring protein that regulates zinc homeostasis (49).

Here, we identify Aspf2 as the first Factor H, FHL-1, and FHR1 binding protein of *A. fumigatus*. Human proteins bound by Aspf2 retain their regulatory activity and inhibit opsonization and phagocytosis by human neutrophils. Also, Aspf2 binds plasminogen, and plasminogen bound to Aspf2 is converted by tissue-type plasminogen activator (tPA) to active plasmin, which cleaves fibrinogen. Furthermore, active plasmin on the surface of *A. fumigatus* conidia damages human lung epithelial cells, induces cell retraction, and matrix exposure.

## Materials and Methods

### Cultivation of *A. fumigatus* Strains

*Aspergillus fumigatus* CEA17 Δ*akuB^Ku80^* (50) (wild type), *A. fumigatus* CEA17 *akuB^Ku80^* Δ*aspf2* mutant, and the Aspf2-complementation strain *A. fumigatus* CEA17 *akuB^Ku80^aspf2^C^* strains were cultivated on *Aspergillus* Minimal Medium (AMM) for 5 days at 37°C in dark, as described (51). Conidia were harvested in sterile deionized water, filtered through 40-µm cell strainers (BD Biosciences, Heidelberg, Germany), washed, resuspended in deionized sterile water, and enumerated with a Thoma counting chamber. Resting conidia of wild type, Δ*aspf2*, and *aspf2^C^* (the complementation strain) were used wherever the conidia used in the experiments.

### Serum, Proteins, and Antisera

Normal human serum (NHS) was collected from healthy individuals upon informed consent, pooled, and stored at −80°C until use. Purified human Factor H, Factor I, C3b, polyclonal goat human C3 antiserum, and polyclonal goat human Factor H antiserum were purchased from CompTech (Complement Technology Inc., Tyler, TX, USA). Serum collection was approved by the Ethical board of the Medical Faculty of the Friedrich Schiller University Jena, Germany. Alexa fluor 488-conjugated donkey anti-goat and Alexa fluor 647-conjugated donkey anti-mouse antibodies were procured from Life Technologies (Darmstadt, Germany). Plasminogen, tPA, or uPA were obtained from Technoclone (Technoclone, GmbH, Vienna, Austria). Polyclonal goat anti-human plasminogen IgGs were obtained from Acris antibodies (Acris antibodies GmbH, Herford, Germany). HRP-conjugated rabbit anti-goat and HRP-conjugated goat anti-mouse were bought from Dako (Dako, Deutschland GmbH, Hamburg, Germany). Monoclonal anti-Aspf2 antibody (clone, 1G12), Factor H (clone: T13), and anti-FHR1 (clone: JHD 7.10) were generated in-house. Mouse anti-Aspf2 antiserum was generated as described previously by immunization of mice with recombinant Aspf2 (52). Recombinant Factor H deletion mutants (SCRs1–4, SCRs1–5, SCRs1–6, SCRs6–8, SCRs8–11, SCRs11–15, SCRs15–18, SCRs15–19, SCRs8–20, SCRs15–20, and SCRs19–20) and FHL-1 were expressed in the baculovirus system as previously described (53). FHR1 and FHR1 deletion mutants SCRs1–2, SCRs3–5 were expressed as described (54). Gelatin was purchased from Merck (Darmstadt, Germany).

### Cloning, Expression, and Purification of Recombinant Aspf2

The gene encoding *A. fumigatus* Aspf2 lacking the N-terminal 19-amino acid spanning signal peptide sequence was cloned into pPICZαB. Therefore, *aspf2* was amplified from cDNA of *A. fumigatus* CEA17 *akuB^KU80^* RNA using primers F2_Pst1_N19_for (5′-actgctgcagccaccctccctacctcccccg-3′) and F2_Xba1_rev (5′-actgtctagattcgagatccg gactgtccttacc-3′), including *Pst*I and *Xba*I restriction sites (underlined). The PCR product was digested with *Pst*I and *Xba*I and ligated into plasmid pPICZαB (Invitrogen, Karlsruhe, Germany). Proper insertion was confirmed by sequencing and the resulting plasmid pPICZαB-ΔN19AspF2 was transformed into *Pichia pastoris* X33 (EasySelect™ Pichia Expression Kit, Invitrogen, Karlsruhe, Germany). Aspf2 expression was induced by 1% (v/v) methanol. After 3 days of induction, the culture supernatant was harvested, and recombinant protein was purified after passing through HisTrap 5-ml column.

### Enzyme-Linked Immunosorbent Assay

Factor H, the Factor H deletion mutants, FHL-1, FHR1, the FHR1 deletion mutants, plasminogen, or gelatin (1 μg/well in 100 µl DPBS) were immobilized onto microtiter plate at 4°C overnight. After blocking with 0.2% (w/v) gelatin, Aspf2 (1 μg/well) was added and the mixture was incubated for 1 h at room temperature (RT). Following washing, mouse anti-Aspf2 antiserum was added followed by horseradish peroxidase-conjugated secondary IgGs. After washing, TMB was added and the reaction was stopped with 2 M H_2_SO_4_. Absorbance signals were measured at 450 nm with Multiskan Ascents (Thermo Labsystems).

### Combined ELISA-Based Western Blotting Analysis (CEWA)

Aspf2 or gelatin (2 μg/well in 100 µl DPBS) were immobilized onto microtiter plates at 4°C overnight. After blocking with 0.2% (w/v) gelatin, purified Factor H, FHL-1 at the indicated concentrations or NHS-EDTA was added. Following incubation, bound proteins were eluted with 1x Roti^®^ Load 1, boiled at 95°C for 10 min, and separated by SDS-PAGE. Proteins were transferred onto a membrane, and Factor H was identified by western blotting using goat anti-human Factor H antiserum.

### Biolayer Interferometry

Binding of Factor H to immobilized Aspf2 was analyzed in real time by biolayer interferometry (Forte Bio, Menlo Park, CA, USA). The Ni(II)-NTA biosensor was loaded with recombinant Aspf2. Before measurement, biosensor tips were briefly washed (30 s) to remove free Aspf2, and Factor H at concentrations from 190, 375, 750, 1,500, and 3,000 nM was added as an analyte. For each concentration, the association of Factor H to Aspf2 was followed and after removal of the analyte the dissociation of the complexes was followed for 150 s. Denatured Factor H (95°C for 10 min) was used as a non-binding control.

Binding of plasminogen to Aspf2 was determined by adding plasminogen at 687.5, 1370, 2,750, and 5,500 nM as an analyte. For each concentration, the association and dissociation of plasminogen to Aspf2 was followed for 200 s. The *K*_d_ values were determined by subtracting the values from buffer control, using the BLITZ software. The association and dissociation curves were plotted using Graphpad5.

### Cofactor Assay

To demonstrate the cofactor activity of Factor H or FHL-1 bound to Aspf2, the human regulators were bound to immobilized Aspf2 (2 μg/100 μl). After blocking for 1 h at RT, Factor H [0.5–4 µg in Hanks balanced salt solution (HBSS) with Ca^2+^ and Mg^2+^] was added for 1 h at RT. In addition NHS at the indicated concentrations was added to immobilized Aspf2 for 1 h at 37°C. After washing, C3b (1 µg/well) and Factor I (1 µg/ml) were added and following 1 h incubation at 37°C, the reaction was stopped and the proteins were separated by SDS-PAGE. Proteins were transferred to a membrane and C3b degradation products were visualized by goat anti-human C3 antiserum, followed by an HRP-conjugated rabbit anti-goat antibody.

### Indirect Immunofluorescence Assay for Factor H, FHL-1, and Aspf2

*Aspergillus fumigatus* conidia (5 × 10^6^ cells/ml) were allowed to adhere to coverslips pre-coated with poly-l-lysine for 1 h at RT. For swollen conidia and hyphae induction, the resting conidia were incubated for 4 and 8 h, respectively, at 37°C in RPMI 1640 with 10% (v/v) FCS. The non-adherent cells were washed off, and the adherent cells were fixed in 4% paraformaldehyde (PFA) in PBS for 10 min. After washing, auto-fluorescence was quenched with ammonium chloride (150 mM) for 10 min at RT. The cells were blocked with 3% (w/v) BSA in DPBS and Factor H (40 µg/ml) or FHL-1 (20 µg/ml) were added for 1 h at RT. Following washing, goat anti-human Factor H antiserum was added in combination with mouse anti-Aspf2 antiserum in blocking buffer for 1 h at RT, followed by corresponding Alexa fluor fluorochrome-conjugated secondary IgGs. After washing, the coverslips were mounted on glass slides with Mount Fluor (Pro Taqs), and images were obtained with LSM 710 (Zeiss, Germany) using ZEN 2009 software.

### Generation of *A. fumigatus aspf2* Knockout and Complementation Strain

*Aspergillus fumigatus* CEA17 *akuB^KU80^* (50) was used as a parental strain for the generation of an *aspf2* knockout mutant using a 3-fragment-fusion-PCR-based gene disruption approach. The upstream (left flank) and downstream (right flank) coding regions of *aspf2* were amplified from chromosomal DNA of *A. fumigatus* by PCR using primers F2-LF_for (5′-tcgggcagcgacagttttacgtgc-3′) and F2_LF_ptrA_rev(5′-ggcctgagtggccatcgaattcttgtgagtacattttctttttcaagg-3′) and F2_RF_ptrA_for (5′-gaggccatctaggccatcaagcgagggatggaaatgactgacgttgg-3′)/F2_RF_rev (5′-tctcatttcccgcgtgttacaagg-3′), respectively. The pyrithiamine resistance cassette was amplified from plasmid pSK275 with the primer pair ptrA_for (5′-gaattcgatggccactcaggccaattg-3′)/ptrA_rev (5′-gcttgatggcctagatggcctcttgcatc-3′) (55). The *aspf2* deletion construct was generated by overlap extension PCR using primers F2-LF_for and F2_RF_rev (55). The synthesized linear 3-fragment fusion DNA product comprising the 1.5-kb upstream and downstream coding regions of Aspf2 (AFUA_4G09580) flanking the pyrithiamine resistance cassette was transformed and the transformants were selected on AMM agar plates supplemented with 0.1 µg/ml pyrithiamine (Sigma, Germany) as described (51).

*Aspergillus fumigatus* CEA17 *akuB^KU80^* Δ*aspf2* was used as a parental strain for the generation of an *aspf2*-complementation mutant using a 3-fragment-fusion-PCR approach. The upstream (left flanking region) and the coding region of *aspf2* were amplified from chromosomal DNA of *A. fumigatus* CEA17 *akuB^KU80^* by PCR using primers F2_LF_for (5′-tcgggcagcgacagttttacgtgc-3′) and aspf2_rev (5′-ctaagtgcaatgaagctgtc-3′), while the downstream (right flanking region) aspf2 was amplified using primers F2_RF_ for (5′-gaggccatctaggccatcaagcgagggatggaaatgactgacgttgg-3′) and F2_RF_rev (5′-tctcatttcccgcgtgttacaagg-3′). The *A. oryzae* hygromycin (*hph)* cassette was used as a selectable marker and was amplified with the primer pair Ttef_Aspf2tail_for (5′- catgaaggtggacagcttcattgcacttaggcggacattcgatttatgcc-3′)/hph_RFtail_rev (5′- gcatacggtcataaaatatcgtgtcctcgcctattcctttgccctcggac -3′). The *aspf2* deletion construct was generated by overlap extension PCR using primers F2-LF_for and F2_RF rev (55). The synthesized linear 3-fragment fusion DNA product comprising the 0.8-kb upstream coding region of the gene aspf2, the gene aspf2 (AFUA_4G09580), the hygromycin resistance cassette and the downstream 1-kb coding region of gene aspf2 was transformed, and the transformants were selected on AMM agar plates supplemented with 150 µg/ml hygromycin (Invivogen, Toulouse, France).

Deletion and subsequent reconstitution of the *aspf2* gene were confirmed by Southern blotting. Genomic DNA was isolated from the transformants and the wild-type strain, and digested with BamH1. The digested DNA was separated on a 1% (w/v) agarose gel and blotted onto a Hybond™-N^+^ membrane (GE Healthcare Biosciences, Freiburg, Germany) by capillary blotting. The digoxigenin-labeled (Roche Applied Science, Mannheim, Germany) upstream left flank coding region of *aspf2* served as a hybridization probe. For hybridization of the DNA and detection, the DIG Easy Hyb and the CDP-*Star* ready-to-use kit (Roche Applied Science, Mannheim, Germany) were used according to the manufacturer’s instructions. Signals were detected using a Fusion FX-7 (Vilber Lourmat, Marne La Vallée, France).

### Testing Growth Susceptibility

Growth susceptibility assays were performed as previously described (56). In short, freshly harvested spores from wild-type and Δ*aspf2* strains were serially diluted to concentrations of 10^5^, 10^4^, 10^3^, and 10^2^ conidia. Conidial suspensions (5 µl) were spotted on AMM agar supplemented with various antifungal drugs and stressors. High-glycerol osmolarity (HOG) pathway was tested using KCl (0.6 M). Cell wall integrity was assessed by addition of cell wall damage compounds Congo Red (20 mg/l) or Calcofluor white (30 mg/l). Membrane integrity was tested by supplementation with SDS (0.007%, w/v). The antifungal sensitivity was assessed using azole and the antifungal compounds Amphotericin B (2.5 µg/ml), Caspofungin (0.1 mg/ml), Itaconazole (0.125 µg/ml), and Terbinafine (0.5 µg/ml). Agar plates were cultivated at normoxic conditions at 37°C for 48 h and growth was subsequently documented.

### Flow Cytometry

To determine the binding of complement regulators to the surface of conidia by flow cytometry, conidia from the wild-type and Δ*aspf2* strain (10 × 10^6^/well) were first treated with BSA (3%, w/v) for 30 min at RT. After washing, conidia were incubated with increasing amounts of purified Factor H, FHL-1, FHR1, plasminogen, or 400 µl NHS-EDTA (10 mM) for 1 h at RT. Following washing, conidia-bound Factor H or FHL-1 was detected with mAB T13 or B22/16 mAbs. FHR1 binding was assessed with JHD 7.10 mAb, and plasminogen binding with goat anti-human plasminogen antiserum. The fluorescence intensities of 10,000 conidia were measured by flow cytometry (BD LSR II), and median fluorescence intensity was calculated with Flowjo.

### Activation of Plasminogen and Plasmin Cleavage Assays

Aspf2 (1 µg/well) was immobilized on the surface of a microtiter plate. After blocking, plasminogen (1 µg/well) was added, followed by tPA (10 ng/well) and the substrate S-2251. Plasmin-mediated cleavage of the substrate S-2251 was determined by recording the absorbance at 405 nm (SpektraMax 190; Molecular Devices).

### Influence of ϵ-aminocaproic acid (ɛ-ACA) and Lysine Residues on Aspf2 Binding to Plasminogen

Increasing concentrations of ε-ACA, lysine, arginine, and glutamic acid (0.3125, 0.625, 1.25, 2.5, and 5 mM) were pre-incubated plasminogen (10 µg/ml) for 1 h at RT. After incubation, the complex was added to immobilized Aspf2 for 1 h, and then the bound plasminogen was determined with HRP-conjugated anti-plasminogen antibody.

### Plasmin-Mediated Fibrinogen Degradation

Plasminogen (2 µg/well) was added to immobilized Aspf2 (1 µg/well), and then uPA (0.1 µg/well, Technoclone) and fibrinogen (2 µg/well, Corning) were added. Following incubation at 37°C, the reaction was stopped at different time points by addition of Roti-Load 1. Then the proteins were separated by SDS-PAGE and transferred to a membrane. Fibrinogen degradation was identified by Western blotting using rabbit anti-human fibrinogen antiserum (Sigma).

### C3b Deposition and Phagocytosis

*Aspergillus fumigatus* wild-type, Δ*aspf2, and aspf2^C^* conidia (5 × 10^6^) were treated with BSA (3%, w/v) for 30 min at RT and then challenged with NHS (20%, v/v; in 20 mM HEPES, pH 7.4, 144 mM NaCl, 7 mM MgCl_2_, 10 mM/EGTA) for 20 min at 37°C. After washing, surface-deposited C3b was detected with goat anti-human C3 anti-serum, followed by Alexa-647-conjugated donkey anti-goat IgG for 1 h at RT. Following washing, the fluorescence intensity was analyzed on a flow cytometer and median fluorescence intensities were calculated. For phagocytosis and killing of conidia by polymorphonuclear granulocytes (neutrophils), human neutrophils were isolated as previously described (57). Neutrophils were isolated from fresh venous blood of healthy adult volunteers withdrawn according to a protocol approved by the Institutional Review Board of the Jena University Hospital (approval number: 2395-10/08 and 5074-02/17) in agreement with the Declaration of Helsinki. The FITC-conjugated wild-type, Δ*aspf2, and aspf2^C^* conidia (5 × 10^6^) were challenged with complement-active human serum NHS, heat-inactivated inactive human serum NHS (hiNHS) (20%, v/v, in 20 mM Hepes pH 7.4, 144 mM NaCl, 7 mM MgCl_2_, 10 mM/EGTA) or buffer (HBSS) in the presence of freshly isolated neutrophils (1 × 10^6^) (5:1 ratio) for 25 min at 37°C. Then neutrophils with phagocytized conidia were identified based on granularity and FITC positivity by flow cytometry (LSR II, BD Biosciences).

### Neutrophil Killing Assay

To investigate the impact of Aspf2 expression on fungal survival, freshly isolated neutrophils (2 × 10^6^) were coincubated with wild-type, Δ*aspf2*, and *aspf2^C^* conidia in multiplicity of infection PMNs: conidia = 1:2. Cells were coincubated for 5 h at 37°C, 5% CO_2_ in 200 µl phenol-free RPMI 1640 medium (Gibco, Life Technologies), supplemented with 5% (v/v) complement-active NHS on 96-well plates (Grenier Bio-One, Austria). At the end of the coincubation time, samples were scraped off the plates and transferred to sterile 1.5 ml tubes. PMNs were lysed with 800 µl sterile deionized ice-cold water for 10 min with rigorous vortexing. Samples were then diluted sequentially to a final dilution of 1:10,000 in PBS (Lonza, Switzerland) containing 0.5% (v/v) Tween 20 (Carl Roth, Germany). Twenty microliters of the final dilutions were plated on Sabouraud 2% (w/v) glucose-agar (Carl Roth, Germany) plates (Ø 145 mm × 20 mm, Grenier Bio-One, Austria). Plates were incubated for 48 h at 37°C and colony forming units were counted.

### Cell Viability and Quantification of Cell Retraction

Human lung epithelial cells A549 (ACC 107) were cultivated in DMEM supplemented with fetal calf serum (FCS, 10%, v/v) at 37°C in humidified 5% (v/v) CO_2_ incubator. A549 cells were seeded (5 × 10^4^ cells/well) in a 96-well plate and grown to confluence. To investigate how plasmin bound to conidia affects epithelial cells, conidia (5 × 10^6^/well) were fixed with 4% PFA for 10 min at RT. After washing, conidia were incubated with plasminogen (100 µg/ml) for 1 h at RT. Following washing, the plasminogen-labeled conidia were added to A549 cells together with tPA (50 µg/ml) in FCS-free DMEM. Cells were then pelleted (200 × *g* for 1 min). After incubation at 37°C at 5% (v/v) CO_2_ for 4 h, FCS (10 µl) was added to each well, and the cells were further incubated at 37 C° overnight. Following removal of unbound conidia, cell titer-blue (100 µl) (Promega GmbH, Mannheim, Germany) was added and the cells were again incubated at 37°C for 2 h. Generation of resorufin was measured at 570 nm using 600 nm as a reference.

To monitor epithelial cell retraction, A549 cells were plated (5 × 10^5^ cells/well) on coverslips in a 24-well plate and grown to confluence. Then plasminogen-bound conidia (25 × 10^6^/well) were added to the cells together with tPA (100 µg/ml) in FCS-free DMEM, and the cells were incubated at 37°C at 5% (v/v) CO_2_ for 2 h. In addition, the serine protease inhibitor aprotinin was added (100 µg/ml). After removal of unbound conidia, A549 cells were fixed with 4% PFA, cells were stained with DAPI (10 µg/ml) and Texas-Red conjugated Wheat-Germ-Agglutinin (10 µg/ml, Thermo Fisher Scientific). The coverslips were mounted on the glass slide and images were acquired with LSM 710 (Zeiss, Germany). The acquired images were used for the quantification of the number of extracellular gaps formed and the area of exposed surface matrix as described previously with bioinformatics image analysis (58).

The morphology and cell retraction was evaluated by scanning electron microscopy (SEM). Again plasminogen-coated conidia (25 × 10^6^/well) were added to A549 epithelial cells in FCS-free DMEM. After incubation, unbound conidia were removed in 0.1 M cacodylate buffer (pH 7.2) (Sigma-Aldrich), and both bound conidia and human epithelial cells were fixed with glutaraldehyde/cacodylate buffer (2.5%, v/v) (Carl Roth, GmbH) for 1 h at RT. After washing, cells were treated with osmium tetroxide/cacodylate (1%, v/v) and dehydrated by adding increasing concentrations of ethanol (10–96%, v/v) for 10 min, followed by critical point-drying in a Leica EM CPD300 Automated Critical Point Dryer (Leica, Wetzlar, Germany). Then, the cover slips were mounted on the aluminum sample holders (stubs) and gold sputter coated (layer thickness 20 nm) in a BAL-TEC SCD 005 Sputter Coater (BAL-TEC, Liechtenstein). Electron images were acquired with a Zeiss (LEO) 1530 Gemini field emission scanning electron microscope (Zeiss, Germany) at 8 kV acceleration voltage and a working distance of 5–8 mm with an Inlense secondary electron detector.

Cell retraction in electron microscopic images was quantified by automated image analysis. An algorithm was written in the Python programming language (version 2.7.12) combined with the openCV (version 2.4.8) (59) and NumPy [version 1.12.0, part of the SciPy package (60)] libraries. The algorithm uses a Gaussian Mixture Model (GMM) trained on the local variance of pixel intensities to distinguish between the two classes of pixels: (i) pixels in areas containing cells and (ii) pixels in areas of the exposed surface matrix. This approach is similar to the segmentation of immune cells in bright field images (61, 62). The algorithm uses the textural differences between the smooth matrix surface and the roughly textured cell layer. All images were preprocessed by applying intensity normalization, followed by noise filtering using a Gaussian filter with σ = 0.5 px and a kernel of 3 × 3 px. Afterward, the local variance in pixel intensities was computed for each image pixel in a neighborhood of 3 × 3 px. One image from data set 5 (A549 cells + plasminogen-bound conidia + tPA) was used to learn the parameters of the GMM. The trained GMM was then applied to all images to predict the class membership of each pixel. The results of this process are connected pixel regions that represent either the areas of the exposed surface matrix or of the cell layer. Regions of the exposed matrix that were smaller than 80 px were removed to exclude minor cell damage and false positives from further analysis. The contours of the remaining matrix regions were smoothed using morphological closing and opening with an elliptical kernel of 3 × 3 px. The pixel areas of all regions that represent the exposed surface matrix were quantified to estimate the extent of the cell damage.

### Statistical Analysis

Statistical analyses of paired t-tests were performed with Graphpad Prism 5 (GraphPad Inc., La Jolla, CA, USA). Differences with *p* < 0.05 were considered significant, and statistical significance is shown as *p < 0.05, **p < 0.01, ***p < 0.001.

## Results

### Recombinant Aspf2 Binds Factor H and FHL-1

**Figure 1:**
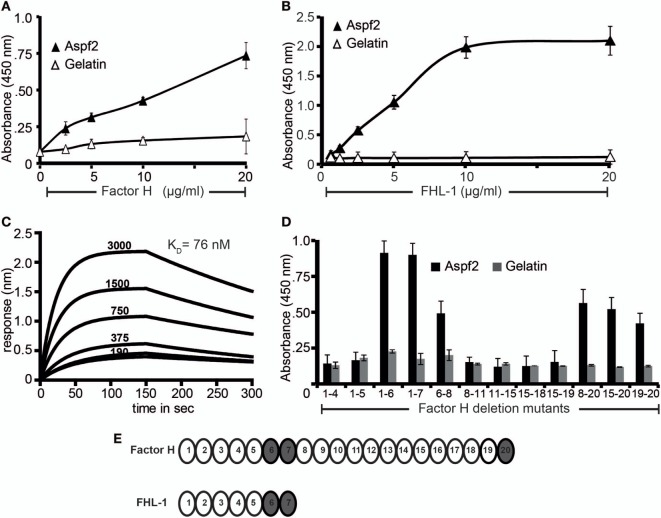
Recombinant Aspf2 binds to Factor H and factor-H-like protein 1 (FHL-1). **(A)** Aspf2 binds to Factor H dose-dependently. Aspf2 binding to Factor H was assayed by enzyme-linked immunosorbent assay (ELISA). Factor H was immobilized onto a microtiter plate, and Aspf2 at the indicated amounts was added. After washing, bound Aspf2 was detected with mAb Aspf2. Aspf2 did not bind to gelatin. **(B)** Aspf2 binds to FHL-1 dose-dependently. Aspf2 at increasing amounts was added to immobilized FHL-1, and bound Aspf2 was detected by with mAb Aspf2. Aspf2 did not bind to gelatin. **(C)** Factor H binds to Aspf2 with high affinity. The binding affinity of Factor H to Aspf2 was evaluated by biolayer interferometry. Aspf2 was coupled to the surface of NTA biosensors, and Factor H at 190, 375, 750, 1,500, and 3,000 nM concentrations was added as analyte. For each concentration, the association of complex was assessed for 150 s, and the dissociation was evaluated for 150 s. Factor H binds to Aspf2 with a K_D_ of 76 nM. Heat-inactivated (95°C) Factor H did not bind to Aspf2 (bottom line) **(D)** Mapping of Aspf2-binding regions within Factor H and FHL-1. Binding of Aspf2 to the indicated Factor H/FHL-1 deletion mutants was investigated by ELISA. Aspf2 was added to immobilized Factor H/FHL-1 deletion mutants, and bound Aspf2 was detected by mouse Aspf2 anti-serum. Aspf2 binds to Factor H *via* SCR6–7 and SCR20 and to FHL-1 through SCR6–7. **(E)** The structure of Factor H and FHL-1 are shown with their corresponding domains short consensus repeats (SCR). Aspf2-binding domains are highlighted in *brown*. Panels A, B, and D represent mean ± SD of three independent experiments.

*Aspergillus fumigatus* recruits the human soluble complement regulators Factor H, FHL-1, and C4BP (22). We, therefore, asked whether Aspf2, the homolog of the *C. albicans* immune regulator binding protein Pra1 (63) also binds Factor H and FHL-1. To this end, Aspf2 was cloned, expressed as a His-tagged protein in *P. pastoris*, and purified to homogeneity (data not shown). Aspf2 bound to both immobilized Factor H and FHL-1, and binding was dose-dependent as assayed by ELISA (Figures 1A,B). Furthermore, the interaction was confirmed in reverse orientation by CEWA. Both Factor H and FHL-1 bound to Aspf2, and binding was dose dependent (Figure S1A in Supplementary Material, upper panel lanes 1–4, FHL1: lower panel lanes 1–4). Binding of Factor H to Aspf2 was also followed in real time, and the affinity was determined using biolayer interferometry. Aspf2 was bound to a biosensor, and Factor H was added as analyte. The association of the complexes was monitored for 150 s. and reached saturation after ca. 70 s. Upon removal of the analyte, the dissociation of the complex was followed for another 150 s, and the complex dissociated at a slow rate. Factor H bound to Aspf2 with high affinity, i.e., *K*_D_ = 76 nM (Figure 1C).

### Mapping of Aspf2-Binding Regions in Factor H and FHL-1

To localize the binding regions in Factor H and FHL-1, binding of Aspf2 to Factor H/FHL-1 SCR deletion mutants, i.e., SCRs1–4, SCRs1–5, SCRs1–6, SCRs1–7, SCRs6–8, SCRs8-11, SCRs11–15, SCRs15-18, SCRs15–19, SCRs8–20, SCRs15–20, and SCRs19–20 was evaluated by ELISA. The name of the Factor H/FHL-1 deletion mutants represents the domains they retain (Figure 1B). Aspf2 bound to the N-terminal Factor H/FHL-1 deletion mutants consisting of SCRs1–6, SCRs1–7, SCRs6–8, and to the C-terminal Factor H deletion mutant SCR20 (Figure 1D). Thus Aspf2 binds FHL-1 *via* SCRs6–7 and binds Factor H *via* two regions, the first positioned in SCRs6–7 is shared with FHL-1, and the second binding region is located in the C-terminus, i.e., SCR20 (Figure 1E).

### Factor H and FHL-1 Bound to Aspf2 Retain Cofactor Activity

**Figure 2:**
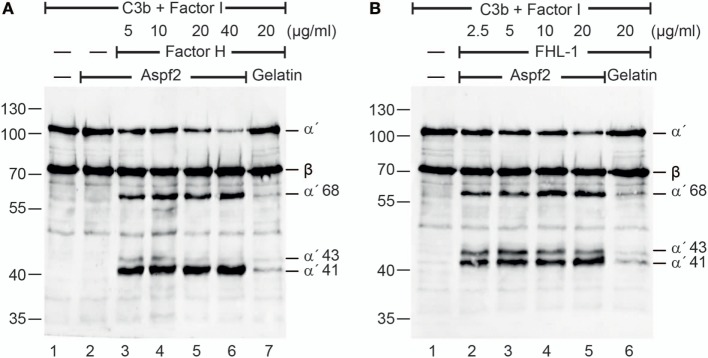
Factor H and factor-H-like protein 1 (FHL-1) bound to Aspf2 retrain cofactor activity. **(A)** Factor H was added at increasing amounts to immobilized Aspf2. After washing, C3b and factor I were added for 60 min; the reaction mixtures were subjected to SDS-PAGE, and C3b cleavage products were visualized by western blotting using goat human C3 antiserum. Factor H bound to Aspf2 assisted in Factor I-mediated cleavage of C3b, as indicated by the appearance of C3b cleavage products α′68, α′43, and α′41 bands (lanes 3–6). Aspf2 alone has no cofactor activity (lane 2). No C3b cleavage was detected in the presence of gelatin (lane 7). **(B)** FHL-1 at the indicated concentrations was added to Aspf2. Followed by C3b and Factor I addition and incubation, the proteins were separated by SDS-PAGE transferred to a membrane and C3 cleavage products were investigated by western blotting. FHL-1 showed cofactor activity and assisted in Factor I-mediated cleavage of C3b, as revealed by appearance of cleavage products α′68, α′43, and α′41 bands (lanes 2–5). These C3b cleavage products were absent in the negative control gelatin (lane 6). Panels **(A,B)** show representative results of three independent experiments.

To determine whether Factor H and FHL-1 bound to Aspf2 maintain regulatory activity, Factor H or FHL1 were bound to immobilized Aspf2, and after washing C3b and Factor I were added. Following incubation for 1 h at 37°C, the supernatants were subjected to SDS-PAGE, and C3b cleavage fragments were identified by western blotting. Factor H when bound to Aspf2 retained cofactor activity, as revealed by the reduced intensity of the C3b α′ chain and the appearance of the α′68, α′43 and α′41 C3b cleavage products (Figure 2A, lanes 3–6). Similarly, FHL-1 bound to Aspf2 assisted in C3b cleavage, as revealed by the appearance of α′68, α′43, and α′41 C3b bands (Figure 2B, lanes 2–5).

Also, serum-derived Factor H and FHL1 when bound to Aspf2 retained cofactor activity for factor I-mediated cleavage of C3b (Figure S2A in Supplementary Material, lanes 2–6). In this case with NHS (10 mM EDTA) was added to immobilized Aspf2, an additional 115 kDa C3 band appeared (Figure S2A in Supplementary Material, lanes 4–6). Given the sequence similarity of Aspf2 with *C. albicans* Pra1, which binds and cleaves C3 (64) we asked whether also Aspf2 binds and cleaves C3 directly. Aspf2 bound to immobilized C3 (data not shown). In contrast to Pra1 from *C. albicans*, Aspf2 did not cleave C3 (Figure S2B in Supplementary Material, lanes 3–4). Thus, Aspf2 binds to C3 but does not cleave this central human complement protein.

We further asked whether Factor H binding to Aspf2 is mediated by C3b, and the proteins form tripartite complexes (65). To this end, Factor H binding from NHS was investigated by CEWA. Factor H derived from NHS (Figure S2C in Supplementary Material, lanes 1–3) or C3 depleted serum bound with similar intensity to Aspf2 (Figure S2C, in Supplementary Material lanes 4–6). Thus, binding of Factor H to Aspf2 was independent of C3b (Figure S2C in Supplementary Material, lanes 4–6).

### Aspf2 Binds the Human Complement Regulator FHR1

Given the similarity of the C-terminal recognition region of Factor H and FHR1, binding of FHR1 was evaluated. Aspf2 bound to immobilized FHR1, and binding was dose dependent (Figure S2D in Supplementary Material). Aspf2 bound to the FHR1 deletion mutant containing only SCRs3–5 but did not bind to the mutant containing only SCRs1–2 (Figures S2E,F in Supplementary Material). The name of the FHR1 deletion mutants, SCRs1–2 and SCRs3–5, represent the domains they retain (Figure S2F in Supplementary Material).

### Generation and Characterization of an *A. fumigatus aspf2* Knockout Mutant

**Figure 3:**
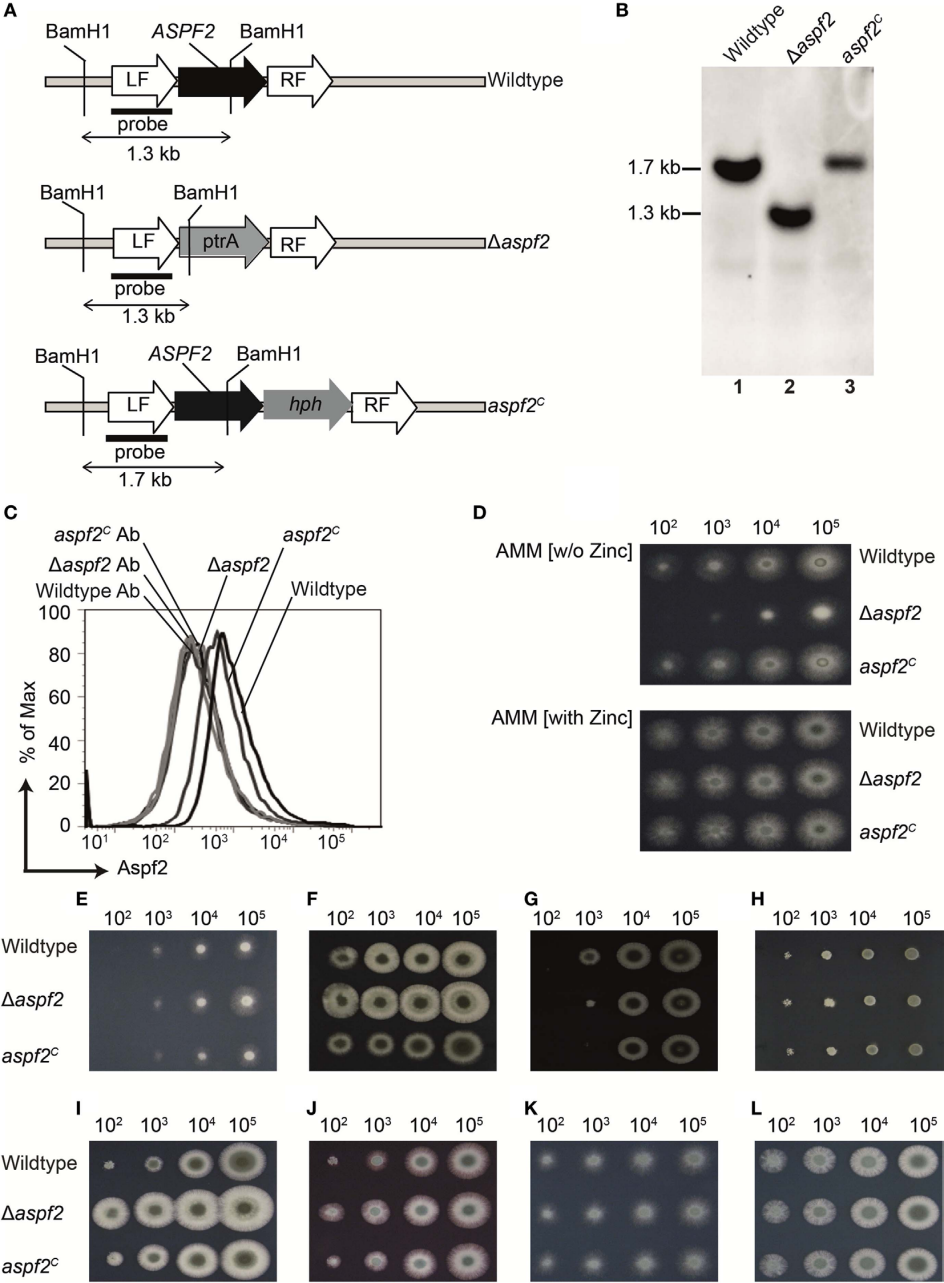
Generation, verification, and phenotypical characterization of *Aspergillus fumigatus* CEA17 *akuB^KU80^*, aspf2 knockout, and Aspf2-complementation strains. **(A)** Schematic overview of the BamH1 restriction sites in the genomic loci of DNA of the wild-type, the Δ*aspf2*, and *aspf2^C^* strains. **(B)** Confirmation of *ASPF2* gene deletion by Southern blotting. Genomic DNA from wild-type and the transformants of Δ*aspf2*, and *aspf2^C^* was digested with BamH1 that has restriction sites in the pyrithiamine resistance cassette (PtrA) present in the gene locus of the mutant strain. Digested DNA fragments were separated on 1% (w/v) agarose gel and blotted onto a membrane that was hybridized with a probe directed specifically against the upstream left flanking region of the *ASPF2* gene. In the Δ*aspf2*, the band characteristic for the wild type (1.7 kbp) disappeared. Instead, a band of 1.3-kbp characteristic for a gene replacement of the *aspf2* locus was detected. As expected, following an in-locus complementation of the knockout strain, the wild-type band at 1.7 kbp was redetected in the *aspf2^C^* strain. **(C)** Expression of Aspf2 on the surface of wild-type, Δ*aspf2, and aspf2^C^* conidia as evaluated by flow cytometry. Aspf2 was absent on the surface of Δ*aspf2* conidia. **(D)** Phenotypical characterization of Δ*aspf2* strains on *Aspergillus* Minimal Medium (AMM) agar in the presence of Zn^2+^ (upper panel) or in the absence of Zn^2+^ (lower panel). Deletion of *ASPF2* gene results in growth defects of the mutant on Zn^2+^-deficient AMM agar. Phenotypic characterization of Δaspf2, wild-type, and aspf2^C^ conidia to various antifungal drugs and stress conditions. Spore suspensions (5 µl) diluted in series were spotted onto AMM agar plates supplemented with Amphotericin B (2.5 µg/ml) **(E)**, Caspofungin (0.1 mg/ml) **(F)**, Itaconazole (0.125 µg/ml) **(G)**, and Terbinafine (0.5 µg/ml) **(H)**. Cell wall integrity was assessed by addition of cell wall damaging compounds Calcofluor white (30 mg/ml) **(I)** or Congo Red (25 mg/ml) **(J)**. High-glycerol osmolarity (HOG) pathway response was tested with 0.6 M KCl **(K)**. Membrane integrity of the conidia was tested upon medium supplementation with SDS (0.007%) **(L)**. Panels **(C**–**E**, **I**–**L)** represent one of three separate experiments. Panels **(E**–**G)** represent one of two separate experiments.

To define how Aspf2 contributes to Factor H- and FHL-1 binding and *A. fumigatus* immune evasion, an *aspf2* knockout strain (Δ*aspf2)* was generated by PCR-based gene disruption (Figure 3A). Successful deletion of the *ASPF2* gene was confirmed by Southern blotting. When probed with the *aspf2* probe, a specific 1.7-kbp band was detected in genomic DNA derived from *A. fumigatus* CEA17 *akuB^KU80^* wild-type strain (Figure 3B, lane 1). The Δ*aspf2* strain lacked this *aspf2*-specific band and a new 1.3 kbp band appeared (Figure 3B, lane 2), thus confirming deletion of the *aspf2* gene. Furthermore, an Aspf2-complementation (*aspf2^C^*) strain was generated by transforming a linear 3-fragment fusion DNA product comprising the 0.8-kb upstream coding region of the gene ASPF2, the hygromycin resistance cassette, and the downstream 1-kb coding region of gene aspf2. The integration of PCR product was confirmed by Southern blotting, and a specific 1.7-kbp band was visible in genomic DNA derived from *aspf2^C^* (Figure 3B, lane 3).

*Aspf2* expression was evaluated by flow cytometry. Aspf2 was expressed by wild-type *A. fumigatus* conidia, and knockout Δ*aspf2* conidia lacked the protein, but *aspf2^C^* conidia expressed Aspf2 on the surface (Figure 3C). Phenotypical analysis of the *aspf2* mutant revealed no differences in germination and growth when conidia were cultivated on AMM, or in rich media such as malt-extract or peptone media (data not shown). The Δ*aspf2* strain did not grow on alkaline and zinc-limiting conditions (Figure 3D, upper panel). However, growth was rescued by zinc supplementation (Figure 3D, lower panel).

We further investigated the role of Aspf2 in response to various antifungal drugs and stress conditions. Deletion of *aspf2* increased resistance to Amphotericin B (Figure 3E), Caspofungin (Figure 3F). At low spore concentrations, the Δ*aspf2* was sensitive to Itraconazole (Figure 3G) but not to Terbinafine (Figure 3H). The Δ*aspf2* strain showed enhanced tolerance to cell wall damaging agents, including Calcofluor white (Figure 3I) and Congo Red (Figure 3J) as compared to wild-type strain. The Δ*aspf2* the wild-type, and the *aspf2^C^* strains showed a similar response to KCl (0.6 M) induced extracellular hyper-osmolarity stress (Figure 3K) and to SDS induced stress (Figure 3L).

### Factor H and Aspf2 (FHL-1 and Aspf2) Colocalize on the Surface of Conidia and Hyphae

**Figure 4:**
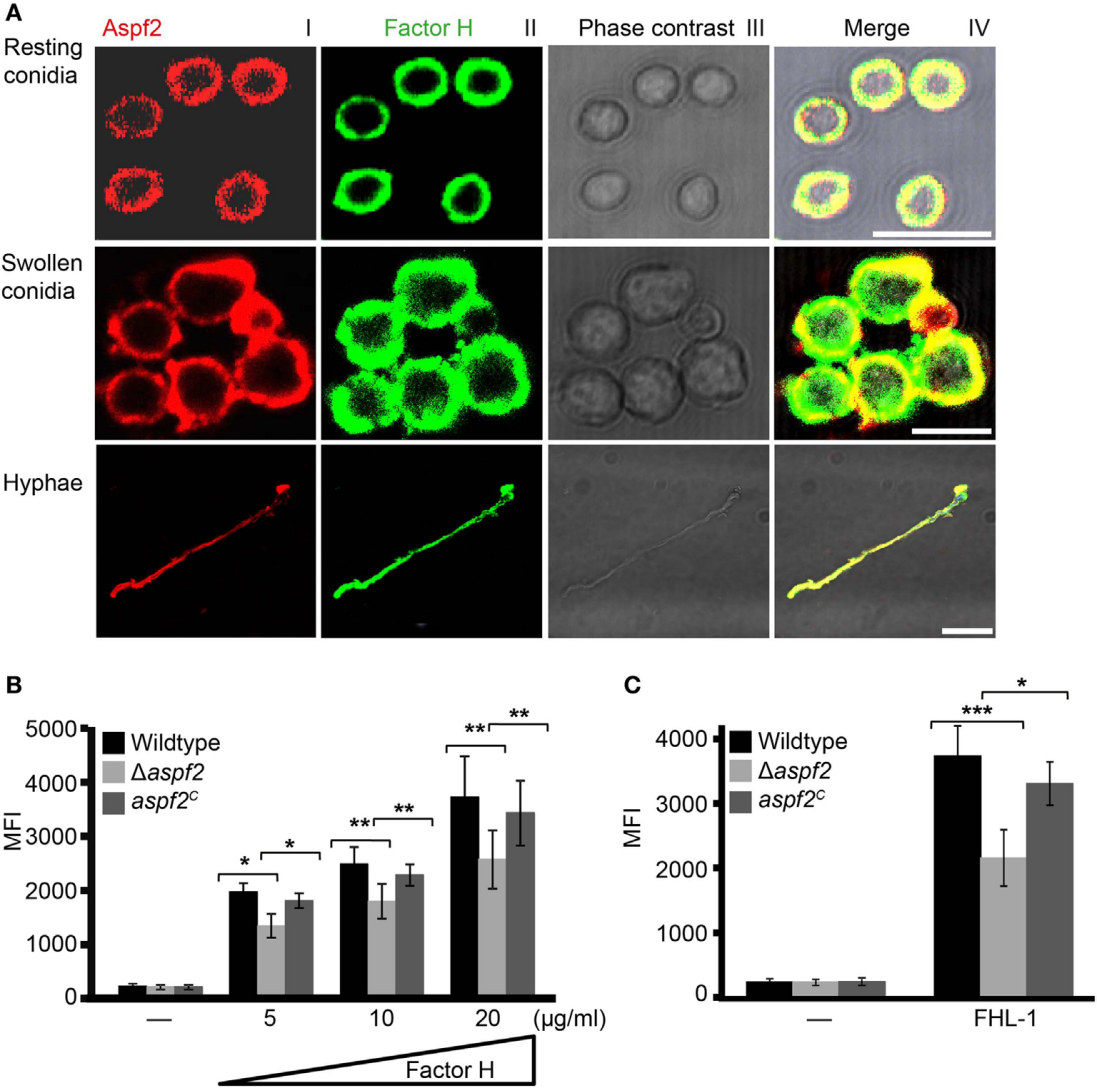
Factor H binds to Aspf2 on the surface of *Aspergillus fumigatus*. **(A)** Factor H and Aspf2 colocalize on the fungal cell surface. Purified Factor H was added to resting conidia, swollen conidia, and hyphae. After washing, the cells were incubated with anti-human Factor H antiserum and mAb Aspf2, followed by corresponding secondary antibodies. Aspf2 and Factor H were colocalized on the surface of resting (upper panel), swollen conidia (middle panel), and hyphae (lower panel). Scale bars 5, 5, and 20 µm in upper, middle and lower panels, respectively. **(B)** Δ*aspf2* conidia bind less Factor H. Δ*aspf2*, wild-type, and *aspf2^C^* conidia were incubated with Factor H at the indicated concentrations, and bound Factor H was investigated by flow cytometry. When Factor H was used at 20 µg/ml, the Δ*aspf2* conidia bound 28% less Factor H as compared to the wild-type and *aspf2^C^* conidia. **(C)** The Δ*aspf2* strain binds factor-H-like protein 1 (FHL-1) with lower intensity. Purified FHL-1 was bound to Δ*aspf2*, wild-type, or *aspf2^C^* conidia and bound FHL-1 was analyzed by flow cytometry. When FHL-1 was used at 10 µg/ml, the Δ*aspf2* strain bound 42% less FHL-1 as wild-type conidia.

Aspf2 distribution on the surface of resting or swollen conidia and hyphae and its contribution to Factor H and FHL-1 binding were evaluated by microscopy. Aspf2 (red fluorescence) was identified on the surface of resting or swollen conidia and hyphae and was evenly distributed on the surface of all three morphotypes, i.e., resting conidia, swollen conidia, and hyphae (Figure 4A; Figure S3A in Supplementary Material). Factor H (green fluorescence) was evenly distributed on the fungal surface, and Factor H and Aspf2 colocalized on the surface of resting conidia, swollen conidia, and on the hyphal surface (Figure 4A). Similarly, FHL-1 (green fluorescence) and Aspf2 colocalized on the surface of the three fungal morphotypes (Figure S3A in Supplementary Material).

### Δ*aspf2* Conidia Bind Factor H and FHL-1 With Lower Intensity

Having shown that Aspf2 recruits Factor H and FHL-1, we asked how conidia surface expressed Aspf2 contributes to regulator binding. Therefore, binding of Factor H or FHL-1 to the conidia was evaluated by flow cytometry. Factor H bound to conidia lacking *aspf2* (Δ*aspf2)*, the wild-type, and Aspf2-complementation conidia (*aspf2^C^*), and this binding was dose dependent. When used at 20 µg/ml, Factor H bound to Δ*aspf2* conidia with about 28% lower intensity (MFI = 2,414 ± 415; mean ± SD), as compared to wild-type Aspf2-expressing conidia (MFI = 3,341 ± 435; mean ± SD; 100%), and Aspf2 expression on the surface of the *aspf2^C^* conidia restored Factor H binding and binding was similar as to the wild-type conidia (MFI = 3,169 ± 362; mean ± SD) (Figure 4B). The bright intensity of Factor H binding to the conidia surface also indicates that swollen conidia express additional Factor H binding proteins (fHbp). Similarly, FHL-1 bound to the Δ*aspf2* strain with 42% lower intensity as compare to the wild-type strain (MFI = 2,158 ± 436 knockout vs. MFI = 3,738 ± 464 wild-type; mean ± SD; *P* < 0.0001), and binding of FHL-1 to *aspf2^C^* strain was similar to wild-type strain (MFI = 3,312 ± 333; mean ± SD; *P* < 0.0012) (Figure 4C). Also NHS derived Factor H bound to Δ*aspf2* with 31% lower intensity (MFI = 2,223 ± 457 Δ*aspf2* vs. MFI = 3,217 ± 534 wild-type; mean ± SD; *P* < 0.0004) (Figure S3B in Supplementary Material). Aspf2 also contributes to FHR1 binding. FHR1 bound to conidia of the Δ*aspf2* with 33% lower intensity (MFI = 1,967 ± 283) as compared to the wild-type Aspf2-expressing conidia (MFI = 2,954 ± 146; mean ± SD; *P* < 0.0041) and to the *aspf2^C^* conidia (MFI = 2,722 ± 223; mean ± SD; *P* < 0.0129) (Figure S3C in Supplementary Material). Thus, Aspf2 expressed on the surface on conidia contributes to the recruitment of Factor H, FHL-1, and FHR1.

### Δ*aspf2* Conidia Are Efficiently Opsonized and Phagocytosed and Killed by Human Neutrophils

**Figure 5:**
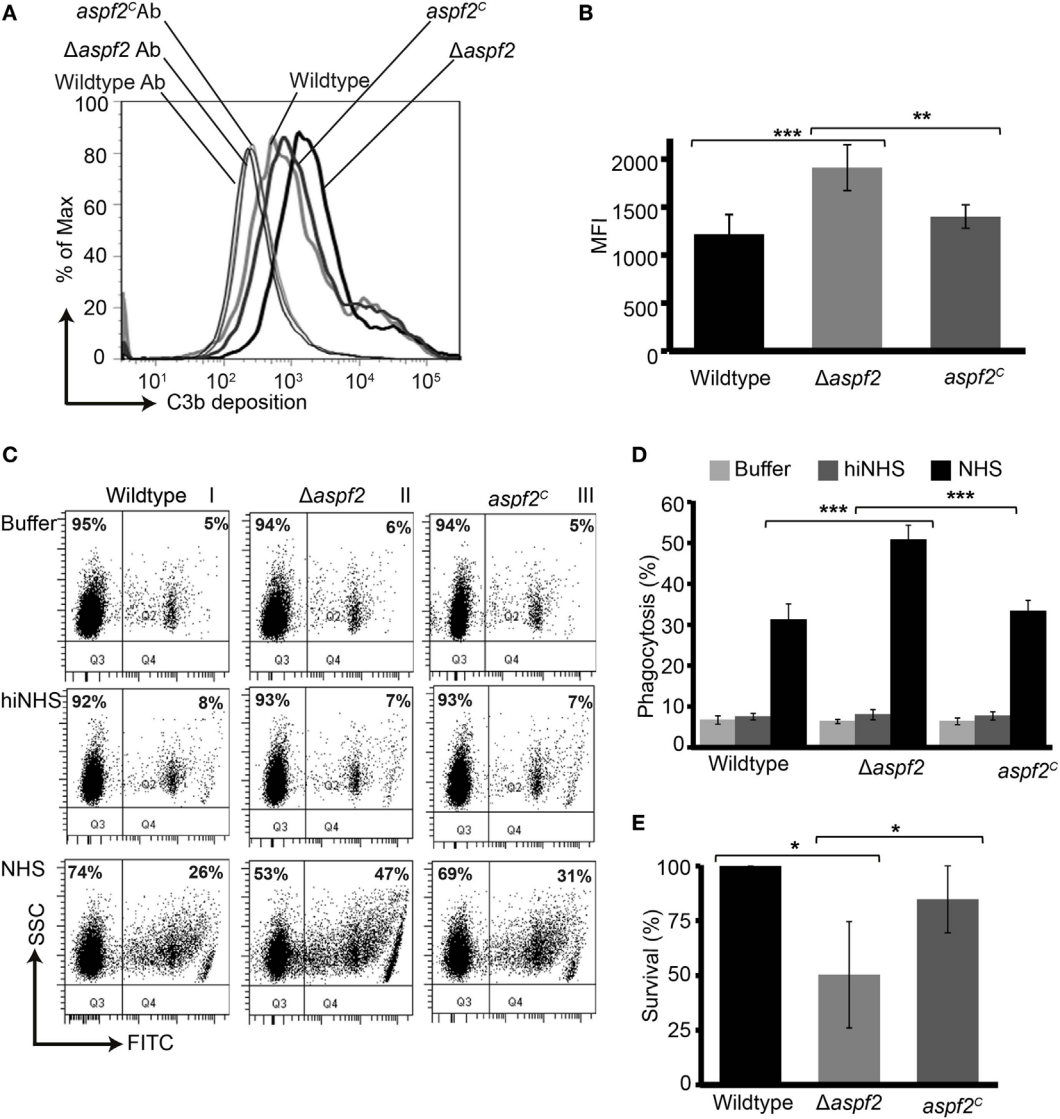
Δ*aspf2* conidia are more efficiently opsonized and phagocytized and killed by human neutrophils. **(A)** Δ*aspf2*, wild-type, and *aspf2^C^* conidia were challenged with complement-active normal human serum (NHS) (20%, v/v) for 20 min. After washing, surface-deposited C3b was analyzed by flow cytometry. **(B)** Δ*aspf2* conidia had more C3b on their surface (157%) as compared to wild-type conidia (100%) and *aspf2^C^* (115%). **(C)** FITC-labeled wild-type, Δ*aspf2*, and *aspf2^C^* conidia were incubated with human neutrophils at 5:1 in the Hanks balanced salt solution (HBSS) (upper panels), or complement inactive hiNHS (middle panels) or in complement-active NHS (20%, v/v) (lower panels) for 25 min. Human neutrophils having conidia phagocytized were identified based on granularity and FITC positivity by flow cytometry (panels I and II, quadrant Q2); neutrophils without conidia were identified as FITC negative cells (panels I and II, quadrant Q1). A representative experiment out of three independent assays is shown. **(D)** In the presence of complement-active human serum (NHS) Δ*aspf2*, conidia were more efficiently phagocytosed (51%) compared to wild-type (31%) and *aspf2^C^* (33%). However, in HBSS (upper panel) or in the absence of complement inactive serum (middle panel), phagocytosis was low and all strains were phagocytosed by the same intensity. Data from **(E)** Δ*aspf2* conidia were efficiently killed by human neutrophils. Δ*aspf2*, wild-type, and Δ*aspf2^c^* conidia were coincubated with human neutrophils for 5 h RPMI 1640 supplemented with 5% (v/v). After incubation, PMNs were lysed with sterile deionized ice-cold water. Samples were then diluted sequentially to a final dilution of 1:10,000 in PBS containing 0.5% (v/v) Tween 20 and plated (20 µl) on Sabouraud 2% (w/v) glucose-agar; colony forming units were counted. The survival of Δ*aspf2* conidia was decreased to 56% compared to wild-type (100%) and *aspf2^C^* conidia (89%). Panels **(B,D,E)** represent mean ± SD of three independent experiments.

As Factor H and FHL-1 bound to Aspf2 retain cofactor activity, we asked how much Aspf2 protects *A. fumigatus* against C3b opsonization. Upon challenge with complement-active NHS, Δ*aspf2* conidia had more C3b deposited on their surface (MFI = 1,909 ± 234; 157%), as compared to wild-type conidia (MFI = 1,214 ± 238; 100%) and Δ*aspf2^C^* conidia (MFI = 1,398 ± 121; 115%) (Figures 5A,B). Higher C3b surface levels correlated with enhanced phagocytoses of FITC-labeled conidia (Figure 5C, lower panel). Δ*aspf2* conidia were more efficiently phagocytosed (FITC positive neutrophils, 47%) as compared to Aspf2-expressin wild-type conidia (26%) and *aspf2^C^* conidia (31%) (Figure 5C). When the data of three independent separate experiment show a similar pattern. Wild-type conidia are more efficiently phagocytosed when incubated in complement-active human serum (bottom panels) as compared to conidia incubate in complement inactive heat-inactivated serum (middle panels) or in buffer (upper panels). Again the aspf2 ko is more efficiently phagocytosed as the wild type and the complementation strain (Figure 5D). Furthermore, Aspf2 contribution to fungal survival was investigated by neutrophil killing. Δ*aspf2* conidia were efficiently killed by human neutrophils, and survival was decreased to 56% compared to wild-type (100%) and *aspf2^C^* conidia (89%) (Figure 5E). Thus Aspf2 contributes to fungal survival.

### Aspf2 Binds to Human Plasminogen

**Figure 6:**
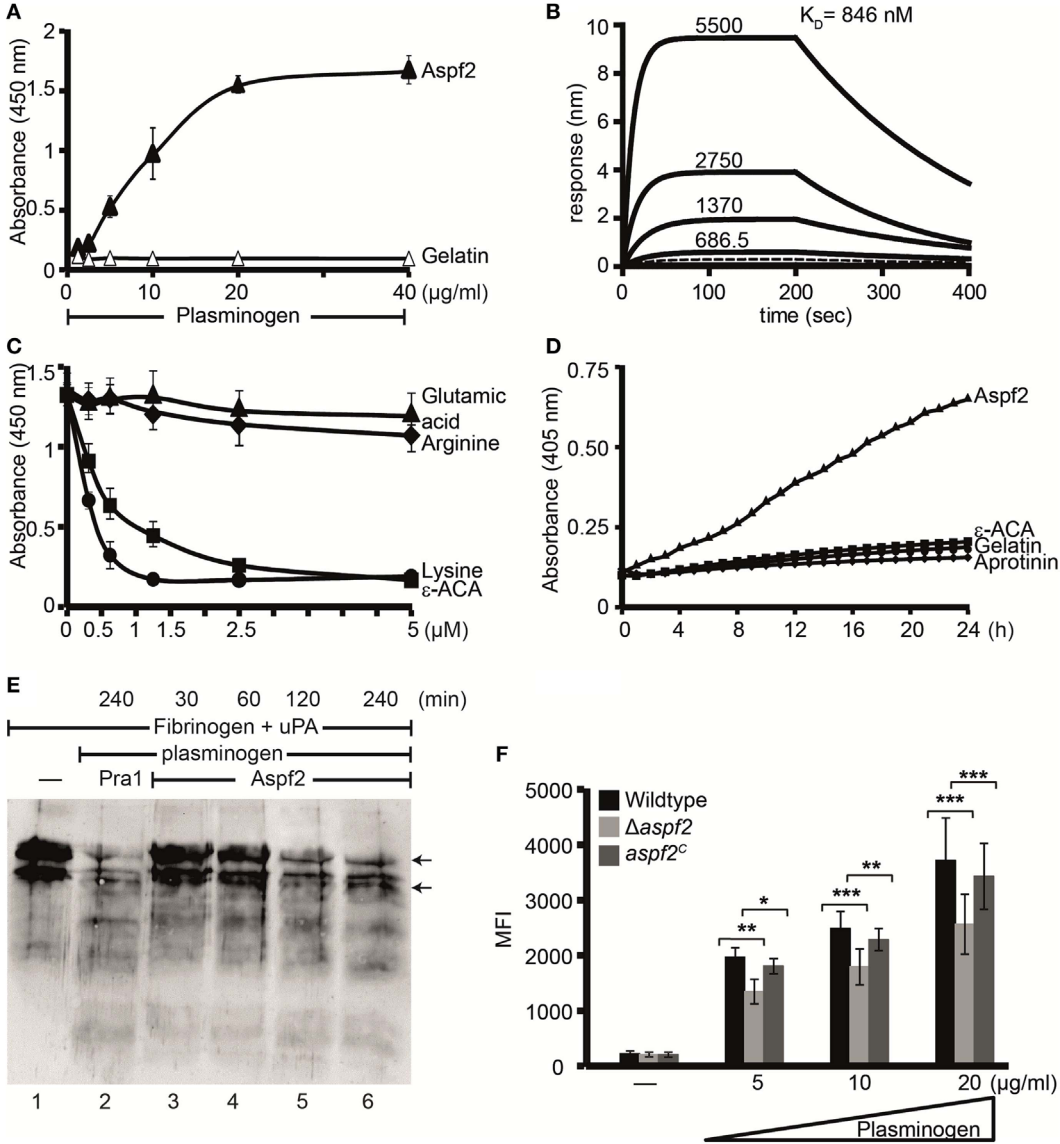
Plasminogen bound to Aspf2 is converted to active plasmin and bound to Δ*aspf2* conidia with less intensity **(A)** Aspf2 binding to plasminogen was assayed by enzyme-linked immunosorbent assay (ELISA). Aspf2 at increasing amounts (0.125–4 µg) was added to immobilized plasminogen, and bound Aspf2 was detected with mouse mAb Aspf2. Aspf2 bound to plasminogen, and binding was dose dependent. Aspf2 did not bind to gelatin. **(B)** Plasminogen binds to Aspf2 with high affinity. The binding affinity of plasminogen to Aspf2 was evaluated by biolayer interferometry. Plasminogen at increasing concentration (687.5, 1370, 2,750, and 5,500 nM) was added to Aspf2 immobilized on Ni-NTA biosensors. For each concentration, the association was evaluated for 200 s. Following removal of the analyte, the complex dissociation was assessed for 200 s. Plasminogen bound to Aspf2 with a *K*_D_ of 846 nM. Heat-inactivated (95°C) plasminogen did not bind to Aspf2 (bottom line). **(C)** The effect of lysine analog and free amino acids on plasminogen binding to Aspf2 was analyzed by ELISA. ϵ-aminocaproic acid (ε-ACA), lysine, arginine, or glutamic acid, at the indicated concentrations, was added to plasminogen, and the mixture was added to immobilized Aspf2. Bound plasminogen was detected with HRP-conjugated anti-plasminogen antibody. ε-ACA and lysine blocked plasminogen binding to Aspf2. Positively charged arginine and negatively charged glutamine did not affect plasminogen binding to Aspf2. **(D)** Plasminogen bound to Aspf2 is converted into functionally active plasmin. Plasminogen was added to Aspf2 immobilized on microtiter plate, followed by urokinase-type plasminogen activator (uPA) and the substrate S-2251. The absorbance was recorded every 30 min over 24 h. Plasminogen when bound to Aspf2 was activated to plasmin which cleaved the chromogenic substrate S-2251 time-dependently. Aprotinin and ε-ACA inhibited cleavage of S-2251. When plasminogen, uPA, and S-2251 were added to immobilized gelatin, S-2251 remained intact and was not cleaved. **(E)** The fibrinolytic activity of Aspf2-bound plasminogen was investigated. Plasminogen was bound to immobilized Aspf2, and after washing, tissue-type plasminogen activator and fibrinogen were added. The reaction was stopped, and the proteins were separated by SDS-PAGE. Degradation of fibrinogen was assessed by Western blotting with a rabbit human fibrinogen antiserum. Plasminogen bound to Aspf2 was converted to plasmin which cleaved fibrinogen (lanes 3–6). When Pra1, a *Candida* immune evasive protein, was used as a positive control, plasmin generated from plasminogen cleaved fibrinogen (lane 2). Fibrinogen (upper arrow) and Fibrinogen degradation products (lower arrow). **(F)** Δ*aspf2* conidia bind less plasminogen. Plasminogen was bound to Δ*aspf2*, wild-type, and *aspf2^C^* conidia. After washing, surface-bound plasminogen was detected by flow cytometry. When plasminogen was used at 20 µg/ml, Δ*aspf2* conidia bound 24% less plasminogen as compared to wild-type and *aspf2^C^* conidia.

Pra1, the *C. albicans* homolog of Aspf2, binds plasminogen (30). Therefore, plasminogen binding to Aspf2 was analyzed. Plasminogen bound to Aspf2, and binding was dose dependent (Figure 6A). When evaluated in real time, plasminogen binding was saturated already after less than 25 s. Plasminogen bound to Aspf2 with a *K*_D_ of 846 nM (Figure 6B). Many microbial proteins attach to human plasminogen *via C*-terminal lysine residues. We, therefore, analyzed if the lysine analog ε-ACA influences binding. ε-ACA inhibited plasminogen binding to Aspf2 and the effect was dose dependent. ε-ACA, used at 1.25 mM, abolished plasminogen binding completely. Also, lysine blocked plasminogen binding, again in a dose-dependent manner. By contrast, neither arginine, nor glutamic acid influenced the plasminogen Aspf2 interaction (Figure 6C). Thus, lysine residues are relevant for plasminogen binding to Aspf2.

### Aspf2-Bound Plasminogen Is Converted to the Active Protease Plasmin

To investigate whether Aspf2-bound plasminogen can be converted to plasmin, plasminogen bound to Aspf2 was treated with uPA, and plasmin activity was assayed. When converted, plasmin cleaved the chromogenic substrate S-2251 and cleavage was time-dependent (Figure 6D). Proteolytic activity was inhibited by the serine protease inhibitor aprotinin and also by ε-ACA. Aspf2-bound plasmin furthermore cleaved the physiological substrate fibrinogen, and this cleavage was time-dependent (Figure 6E, lanes 3–6). Thus, Aspf2-bound plasminogen is converted to the active protease.

### Δ*aspf2* Conidia Bind Less Plasminogen

To define the contribution of Aspf2 for plasminogen binding, binding of plasminogen to wild-type, Δ*aspf2*, and *aspf2^C^* conidia was evaluated. Plasminogen bound to all three strains tested, and binding was dose dependent. Plasminogen (20 µg/ml) binding to Δ*aspf2* conidia was reduced by 31% (MFI 2,563 ± 540) as compared to wild-type conidia (MFI 3,720 ± 761; mean ± SD; *P* < 0.0005). Plasminogen bound to *aspf2^C^* conidia with similar intensity as to wild-type conidia (MFI 3,428 ± 597) (Figure 6F). This remaining plasminogen binding to the knockout mutant is most likely due to the presence of additional plasminogen-binding proteins on the conidial surface.

### Plasmin Bound to Conidia Damages Human Epithelial Cells, induces cell retraction, and matrix exposure

**Figure 7:**
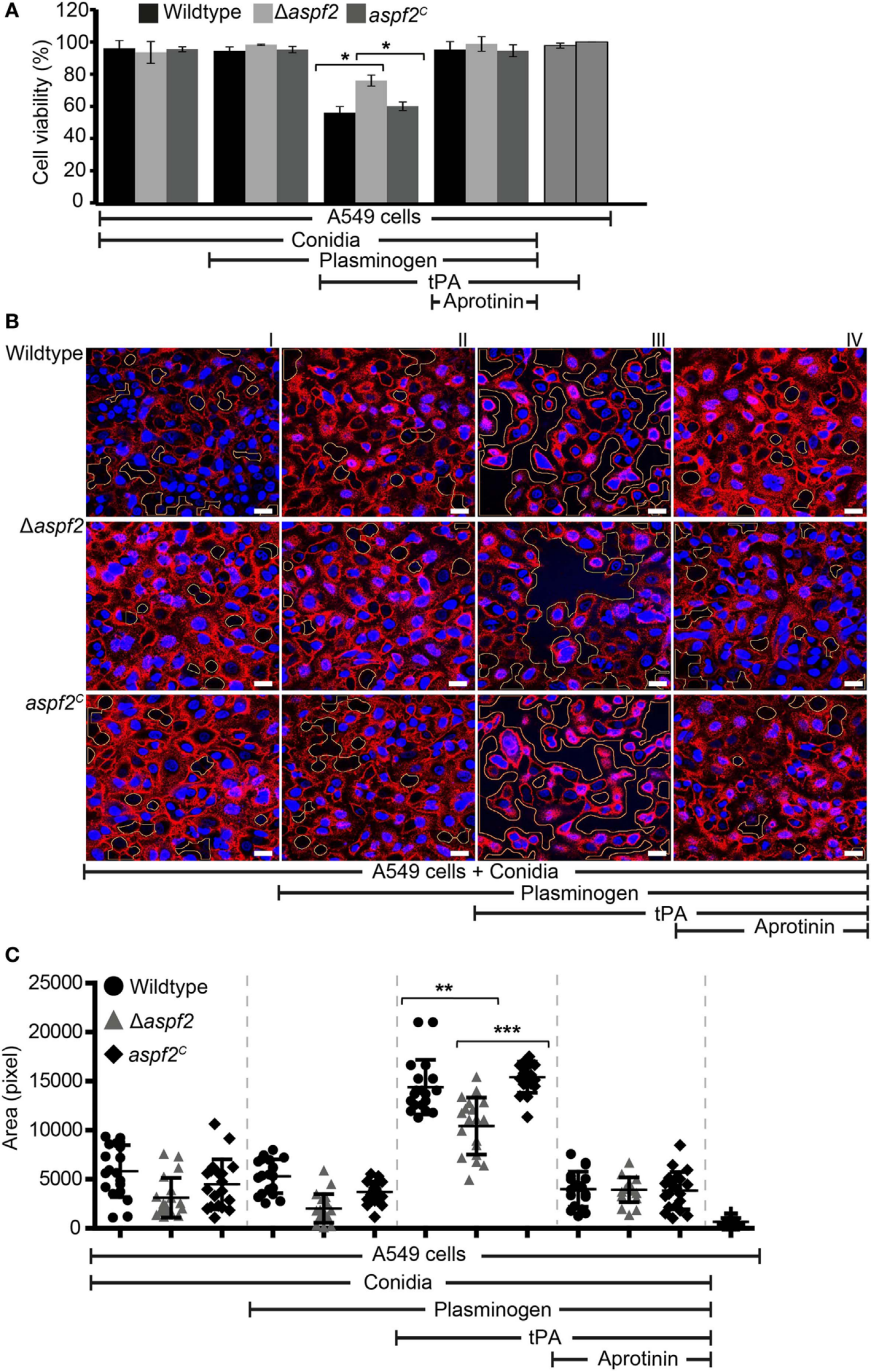
Plasmin bound to conidia damages human lung epithelial cells and induces cell retraction. **(A)** Plasminogen bound to the surface of conidia when activated to plasmin damages human epithelial cells when activated to plasmin. Plasminogen was bound to wild-type, Δ*aspf2*, and *aspf2^C^* conidia. After washing, conidia were added to the cell monolayer in the presence or absence of tissue-type plasminogen activator (tPA). After 24 h unbound conidia were washed off, Cell Titer-blue^®^ was added, and the cellular metabolic activity was followed for 2 h. Plasminogen bound to conidia when converted to active plasmin decreased the metabolic activity of human A549 epithelial cells (columns 7, 8, and 9). This effect was inhibited by the serine protease inhibitor Aprotinin (columns 10, 11, and 12). tPA alone showed no effect on the metabolic activity of A549 cells (columns 13). Viability of A549 cells (columns 14). Panel **(A)** shows mean ± SD of two duplicate independent experiments. **(B)** Active plasmin attached to the surface of conidia induces cell retraction. Plasminogen was bound to wild-type (upper panel) or Δ*aspf2* conidia (middle panel) or *aspf2^C^* conidia (lower panel). After washing, conidia were added to the confluent monolayer of A549 cells together with tPA. After 2 h, unbound conidia were removed; the bound cells were fixed and stained with DAPI (blue) and Texas Red-conjugated Wheat-Germ Agglutinin. Plasminogen bound to conidia when activated to plasmin-induced cell retraction resulting in exposure of extracellular matrix component. Aprotinin blocked plasmin activity. Areas of exposed surface matrix were identified by automated image analysis and are indicated in the images by green lines. Scale bars: 20 µm. **(C)** Bioinformatics quantification of A549 cell damage and cell retraction. Upon evaluating a series of confocal images generated (19 images), the total void area of each image was quantified using bioinformatics image analysis. Active plasmin attached to the surface of wild-type conidia induced cell damage, and the total void area per image was larger as compared to cells damage induced by plasmin attached to the surface of Δ*aspf2* conidia. Plasmin attached to the surface of a*aspf2^C^* conidia induced similar cell damage as compared to the plasmin bound to wild-type conidia. Aprotinin inhibited plasmin activity when attached to wild-type, Δ*aspf2*, and *aspf2^C^* conidia of *Aspergillus fumigatus*. The mean values are indicated by the horizontal red bar, and the SD is shown by the vertical red bars.

Plasmin recruited to the surface of pathogenic microbes cleaves extracellular matrix proteins and damages epithelial cells (58). We, therefore, analyzed whether plasmin attached to the surface of conidia damages human lung cells which belong to the first host cells coming in contact with inhaled *A. fumigatus* conidia. To this end, human A549 lung epithelial cells were challenged with plasminogen-coated conidia. As the first read-out for damage, the cellular metabolic activity of the epithelial cells was quantitated. Plasmin attached to wild-type conidia reduced the metabolic activity of the epithelial cells by about 44% (Figure 7A, column 7). When plasmin was coated to Δ*aspf2* conidia, the metabolic activity was only reduced by 24% (*p* < 0.0013) (Figure 7A, column 8). However, plasmin bound to *aspf2^C^* conidia reduced the metabolic activity of epithelial cells similarly as when bound to wild-type conidia; the metabolic activity was reduced by 40% (Figure 7A, column 9); thus, confirming that Aspf2 contributes to epithelial cell damage. The plasmin mediated cell damage completely blocked by the serine protease inhibitor aprotinin (Figure 7A, columns 10, 11, and 12).

Epithelial cell damage by plasmin-coated conidia was next followed by confocal microscopy. Plasmin attached to wild-type conidia disrupted the cell monolayer, caused cell retraction, and induced exposure of the underlying matrix (Figure 7B, upper row, panel III). This plasmin damage was blocked by aprotinin (Figure 7B, upper row, panel IV). Plasmin attached to Δ*aspf2* conidia caused less damage (Figure 7B, middle row, panel III) compare to the plasmin attached to *aspf2^C^* conidia (Figure 7B, lower row, panel III). Plasmin-induced total void area of each image was quantified by bioinformatics image analysis (58). Plasmin attached to wild-type conidia induced more void area per image (total void area per image; 14,395 ± 2,781 px) as compared to plasmin-attached Δ*aspf2* conidia (total void area per image; 10,418 ± 2,912 px; mean ± SD; *p* < 0.0025; *n* = 19). Plasmin bound *aspf2^C^* conidia induced more total void area per image (total void area per image; 15,185 ± 1,797 px) which similar to the plasmin-attached wild-type conidia (Figure 7C).

Furthermore damage and cell retraction were also evaluated by SEM. Plasmin, when coated to wild-type conidia, caused cell retraction, and a larger void area are formed; the size of the void area was larger (Figure S4A in Supplementary Material, panel II) as when plasmin was bound to Δ*aspf2* conidia (Figure S4A in Supplementary Material, panel III). Multiple SEM images (10 images) were used with a novel bioinformatics image analysis (Figure S4B in Supplementary Material). In this case, the size of the void area was larger with wild-type conidia (surface area per spot: 4,172 ± 11,627) as with Δ*aspf2* conidia (surface area per spot: 437 ± 823; mean ± SD; *P* < 0.0001) (Figure S4C in Supplementary Material). Thus plasminogen recruited to *A. fumigatus* conidia damages human lung epithelial cells and causes matrix exposure. Aspf2 contributes to this plasmin-mediated cell damage as the Δ*aspf2* causes a smaller void area. Thus, Aspf2 contributes to fungal virulence and Aspf2-attached plasminogen when converted to active plasmin can enhance transmission of *A. fumigatus* through lung epithelium.

## Discussion

Fungal pathogens have evolved strategies to combat host immune defense, to cross host tissue barriers, and to colonize at preferred anatomical site. Similar to most microbial pathogens, *A. fumigatus* acquires human plasminogen, which serves several roles in immune evasion. Plasminogen, is activated to the protease plasmin, which degrades the ECM component fibrinogen, controls innate immune attack, and damages lung epithelial cells.

*Aspergillus fumigatus* conidia have been previously shown to specifically bind Factor H and plasminogen (22). However, it remained to be shown which surface proteins of *A. fumigatus* attache these human regulators. Here, we identified Aspf2 as a complement regulator binding protein *of A. fumigatus*, and we showed that Aspf2 is surface exposed and bound several human innate immune regulators. The allergen Aspf2 is the first *A. fumigatus* Factor H and FHL-1 binding protein identified, and Aspf2 also bound plasminogen. Binding of host complement regulators to surfaces of pathogens has emerged as an immune evasion mechanism found for many pathogens (26).

*Aspergillus fumigatus*, the human pathogenic fungus, is predominantly inhaled. Inside the lung, *A. fumigatus* faces a hostile environment and is immediately confronted with host defense proteins. A proteome approach of broncho alveolar lavage and airway surface liquids identified an arsenal of specific host immune defense proteins, including multiple complement components, regulators, plasminogen, recognition proteins, as well as coagulation proteins (66, 67). *A. fumigatus* similar to many other pathogenic microbes is prepared to counteract and control this immune attack.

Here, we identified Aspf2 as a complement regulator protein binding protein *of A. fumigatus*. Aspf2 binds several human innate immune regulators. Aspf2, on the surface of conidia and hyphae, recruited human plasma regulators like Factor H, FHL-1, FHR1, and plasminogen. Factor H and FHL-1, when bound to Aspf2, retained regulatory activity and assisted in the inactivation of C3b. Similarly, plasminogen bound to Aspf2 was activated to the active protease plasmin which degraded ECM component fibrinogen and damaged human lung epithelial cells.

Aspf2 binds both Factor H and plasminogen with nanomolar affinity (*K*_d_—76 and 846 nM, respectively), which are related to the binding affinity of other microbial proteins for Factor H. This Factor H binding affinity is comparable to the Factor H binding to the fHbp of *N. meningitides* which is 5 nM (68). Aspf2-plasminogen binding affinity is similar to the affinity of plasminogen binding to PspC variants of *S. pneumoniae* in the range from 489 to 1,360 nM (58).

Aspf2 attaches Factor H *via* two domains SCR6-7 and SCRs20, and FHL-1 SCR6-7 utilize Factor H and FHL-l for immune protection. These attachment sites in the protein are used by other fungal and microbial pathogens and even by human cells (69). This conserved type of attachment and recruitment shows that pathogenic microbes mimic host cells in exploiting Factor H and the other inhibitors by common mechanisms. These conserved evasion mechanism is indicative that converging evolution is a driving force to develop common immune evasion pathways independently for specific immune evasion (69).

Also plasminogen acquisition is a common evasion mechanism which is used by many microbial pathogens. In addition to Aspf2, *A. fumigatus* enolase, also termed Aspf22, has been previously identified as a plasminogen-binding protein (47). Both Aspf2 and enolase, the moonlighting protein, are surface exposed. Aspf2 and Aspf22 are allergens that induce an IgE antibody response in patients with ABPA (70). Microbial pathogens apparently use several surface proteins, often moonlighting proteins to attach plasminogen. For *C. albicans* more than 10 plasminogen-binding protein have been identified, and many microbial plasminogen ligands also bind Factor H (58). Thus, microbial plasminogen ligands have the ability to attach several host regulators to the microbial surface.

Aspf2 is relevant for immune evasion as the attached host proteins Factor H, FHL-1, and plasmin maintain their regulatory activity, assisted in complement control, damaged human lung epithelial cells, decreased their cellular metabolic activity, induced cell retraction, and caused ECM exposure.

Aspf2 Knockout conidia bound less Factor H, FHL1, and plasminogen as compared to wild-type and *aspf2^C^* conidia. This suggests that *Aspergillus* uses several surface proteins to bind these human regulators. Thus, it will be worthwhile to identify these additional Factor H and plasminogen ligands.

Here, we used a new bioinformatics image analysis which allows quantitating cell damage and cell retraction induced by *A. fumigatus* attached plasmin. This approach allows to count the number of void spots formed by damaged and retracted epithelial cells and to quantitate the void surface area of each individual spot. This novel approach when applied for both confocal images of fluorescently labeled cells and for EM images showed highly related results. Thereby a bioinformatics image analysis extends the information of qualitative microscopic evaluation performed by visual inspection to a quantitative, unbiased evaluation showing both the degree and extends of epithelial cell damage. Thereby, we were able to quantitate epithelial cell damage and to demonstrate that plasmin attached to wild-type conidia more and also larger void spots were formed as when plasmin was attached to the surface of aspf2 knockout conidia. Thus plasmin attached to *A. fumigatus* surface damages human lung epithelial cells, and Aspf2 contributes to epithelial cell damage. These results correlate with recent genetic analyses showing that a SNP in human Plasminogen gene (ID 5340), resulting in exchange of Asp_472_ → Asn, enhanced the risk for invasive aspergillosis among hematopoietic stem cell transplant recipients (1). Thus, plasmin(ogen) adjusts the pathogenesis of invasive fungal infection.

Aspf2 shares 43% amino acid identity with Pra1, the immune evasion protein *C. albicans* (63, 71). Both proteins bind a related set of human plasma regulators and both are zinc chelators (63). In addition, Aspf2 and Pra1 share a metalloprotease-like motif at related positions, Aspf2: **H**_186_**R**LY**H**_190_; Pra1: **H**_178_**R**FW**H**_182_. However, Aspf2 lacks proteolytic activity, but Pra1 of *C. albicans* cleaves human C3 (Figure S1C in Supplementary Material) (64).

In summary, we identify Aspf2 of *A. fumigatus* as a plasminogen binding and complement regulator acquiring surface protein. Aspf2 contributes to fungal immune evasion, as the aspf2 knockout was more efficiently opsonized with C3b and more efficiently phagocytosed. Aspf2 exploits host active protease plasmin which damages human lung epithelial cells, alters the cellular metabolic activity, and induces cell retraction resulting in exposure of the sub-epithelial extracellular matrix which suggest that Aspf2 thereby can enhance fungal invasion into lung tissue. Thus, by assisting in immune evasion and tissue destruction, Aspf2 contributes to the pathogenesis of *A. fumigatus* and represents a new target for immune intervention.

## Ethics Statement

The work was approved by the ethics committee of the Faculty of Medicine, Medical Faculty of the Friedrich Schiller University Jena.

## Author Contributions

Designed experiments: PD, NB, IAS, CS, AAB, and PFZ. Performed experiments: PD, IAS, MS, DW, HM-D, PH; provided research reagents: NB; analyzed data: MW, SD, CS, ZC, MTF, AAB and PFZ; and wrote the manuscript PD, IAS, MTF, ZC, AAB and PFZ.

## Conflict of Interest Statement

The authors declare that the research was conducted in the absence of any commercial or financial relationships that could be construed as a potential conflict of interest.

## Abbreviations

FHL-1, factor-H-like protein 1; FHR1, complement factor H-related protein 1; ELISA, enzyme-linked immunosorbent assay; DPBS, Dulbecco’s phosphate-buffered saline; RT, room temperature; SCRs, short consensus repeats; tPA, tissue-type plasminogen activator; uPA, urokinase-type plasminogen activator; ϵACA, ϵ-aminocaproic acid; CEWA, combined ELISA-based western blotting analysis.

## References

[1] Zaas AK Liao G Chien JW Weinberg C Shore D Giles SS Plasminogen alleles influence susceptibility to invasive aspergillosis. PLoS Genet (2008) 4(6):e1000101. 10.1371/journal.pgen.1000101 18566672 PMC2423485

[2] Dagenais TR Keller NP. Pathogenesis of *Aspergillus fumigatus* in invasive aspergillosis. Clin Microbiol Rev (2009) 22(3):447–65. 10.1128/CMR.00055-08 19597008 PMC2708386

[3] Speth C Rambach G. Complement attack against *Aspergillus* and corresponding evasion mechanisms. Interdiscip Perspect Infect Dis (2012) 2012:463794. 10.1155/2012/463794 22927844 PMC3423931

[4] Kozel TR Wilson MA Farrell TP Levitz SM. Activation of C3 and binding to *Aspergillus fumigatus* conidia and hyphae. Infect Immun (1989) 57(11):3412–7. 2680973 10.1128/iai.57.11.3412-3417.1989 PMC259839

[5] Sturtevant J Latge JP. Participation of complement in the phagocytosis of the conidia of *Aspergillus fumigatus* by human polymorphonuclear cells. J Infect Dis (1992) 166(3):580–6. 10.1093/infdis/166.3.580 1500740

[6] Ma YJ Doni A Hummelshoj T Honore C Bastone A Mantovani A Synergy between ficolin-2 and pentraxin 3 boosts innate immune recognition and complement deposition. J Biol Chem (2009) 284(41):28263–75. 10.1074/jbc.M109.009225 19632990 PMC2788878

[7] Bidula S Sexton DW Abdolrasouli A Shah A Reed A Armstrong-James D The serum opsonin L-ficolin is detected in lungs of human transplant recipients following fungal infections and modulates inflammation and killing of *Aspergillus fumigatus*. J Infect Dis (2015) 212(2):234–46. 10.1093/infdis/jiv027 25612732

[8] Crosdale DJ Poulton KV Ollier WE Thomson W Denning DW. Mannose-binding lectin gene polymorphisms as a susceptibility factor for chronic necrotizing pulmonary aspergillosis. J Infect Dis (2001) 184(5):653–6. 10.1086/322791 11474427

[9] He Q Li H Rui Y Liu L He B Shi Y Pentraxin 3 gene polymorphisms and pulmonary aspergillosis in COPD patients. Clin Infect Dis (2017) 66(2):261–7. 10.1093/cid/cix749 29020397

[10] Hector RF Yee E Collins MS. Use of DBA/2N mice in models of systemic candidiasis and pulmonary and systemic aspergillosis. Infect Immun (1990) 58(5):1476–8. 2323826 10.1128/iai.58.5.1476-1478.1990 PMC258651

[11] Heinekamp T Schmidt H Lapp K Pahtz V Shopova I Koster-Eiserfunke N Interference of *Aspergillus fumigatus* with the immune response. Semin Immunopathol (2015) 37(2):141–52. 10.1007/s00281-014-0465-1 25404120 PMC4326658

[12] Zipfel PF Mihlan M Skerka C The alternative pathway of complement: a pattern recognition system. Adv Exp Med Biol (2007) 598:80–92. 10.1007/978-0-387-71767-8_7 17892206

[13] Mihlan M Stippa S Jozsi M Zipfel PF. Monomeric CRP contributes to complement control in fluid phase and on cellular surfaces and increases phagocytosis by recruiting factor H. Cell Death Differ (2009) 16(12):1630–40. 10.1038/cdd.2009.103 19680263

[14] Hein E Garred P. The lectin pathway of complement and biocompatibility. Adv Exp Med Biol (2015) 865:77–92. 10.1007/978-3-319-18603-0_5 26306444

[15] Malmsten M Schmidtchen A Antimicrobial C3a – biology, biophysics, and evolution. Adv Exp Med Biol (2007) 598:141–58. 10.1007/978-0-387-71767-8_11 17892210

[16] Pasupuleti M Walse B Nordahl EA Morgelin M Malmsten M Schmidtchen A. Preservation of antimicrobial properties of complement peptide C3a, from invertebrates to humans. J Biol Chem (2007) 282(4):2520–8. 10.1074/jbc.M607848200 17132627

[17] Walport MJ Complement. Second of two parts. N Engl J Med (2001) 344(15):1140–4. 10.1056/NEJM200104123441506 11297706

[18] Zipfel PF Skerka C. Complement regulators and inhibitory proteins. Nat Rev Immunol (2009) 9(10):729–40. 10.1038/nri2620 19730437

[19] Merle NS Church SE Fremeaux-Bacchi V Roumenina LT. Complement system part I – molecular mechanisms of activation and regulation. Front Immunol (2015) 6:262. 10.3389/fimmu.2015.00262 26082779 PMC4451739

[20] Skerka C Chen Q Fremeaux-Bacchi V Roumenina LT Complement factor H related proteins (CFHRs). Mol Immunol (2013) 56(3):170–80. 10.1016/j.molimm.2013.06.001 23830046

[21] Heinen S Hartmann A Lauer N Wiehl U Dahse HM Schirmer S Factor H-related protein 1 (CFHR-1) inhibits complement C5 convertase activity and terminal complex formation. Blood (2009) 114(12):2439–47. 10.1182/blood-2009-02-205641 19528535

[22] Behnsen J Hartmann A Schmaler J Gehrke A Brakhage AA Zipfel PF. The opportunistic human pathogenic fungus *Aspergillus fumigatus* evades the host complement system. Infect Immun (2008) 76(2):820–7. 10.1128/IAI.01037-07 18039838 PMC2223477

[23] Behnsen J Lessing F Schindler S Wartenberg D Jacobsen ID Thoen M Secreted *Aspergillus fumigatus* protease Alp1 degrades human complement proteins C3, C4, and C5. Infect Immun (2010) 78(8):3585–94. 10.1128/IAI.01353-09 20498262 PMC2916278

[24] Lesiak-Markowicz I Vogl G Schwarzmuller T Speth C Lass-Florl C Dierich MP *Candida albicans* Hgt1p, a multifunctional evasion molecule: complement inhibitor, CR3 analogue, and human immunodeficiency virus-binding molecule. J Infect Dis (2011) 204(5):802–9. 10.1093/infdis/jir455 21844307 PMC5654502

[25] Vogl G Lesiak I Jensen DB Perkhofer S Eck R Speth C Immune evasion by acquisition of complement inhibitors: the mould *Aspergillus* binds both factor H and C4b binding protein. Mol Immunol (2008) 45(5):1485–93. 10.1016/j.molimm.2007.08.011 17915330 PMC5654503

[26] Zipfel PF Hallstrom T Riesbeck K Human complement control and complement evasion by pathogenic microbes – tipping the balance. Mol Immunol (2013) 56(3):152–60. 10.1016/j.molimm.2013.05.222 23810413

[27] Schmidt CQ Kennedy AT Tham WH. More than just immune evasion: hijacking complement by *Plasmodium falciparum*. Mol Immunol (2015) 67(1):71–84. 10.1016/j.molimm.2015.03.006 25816986

[28] Bennett KM Rooijakkers SH Gorham RD Jr. Let’s tie the knot: marriage of complement and adaptive immunity in pathogen evasion, for better or worse. Front Microbiol (2017) 8:89. 10.3389/fmicb.2017.00089 28197139 PMC5281603

[29] Jozsi M. Factor H family proteins in complement evasion of microorganisms. Front Immunol (2017) 8:571. 10.3389/fimmu.2017.00571 28572805 PMC5435753

[30] Luo S Poltermann S Kunert A Rupp S Zipfel PF. Immune evasion of the human pathogenic yeast *Candida albicans*: Pra1 is a factor H, FHL-1 and plasminogen binding surface protein. Mol Immunol (2009) 47(2–3):541–50. 10.1016/j.molimm.2009.07.017 19850343

[31] Luo S Blom AM Rupp S Hipler UC Hube B Skerka C The pH-regulated antigen 1 of *Candida albicans* binds the human complement inhibitor C4b-binding protein and mediates fungal complement evasion. J Biol Chem (2011) 286(10):8021–9. 10.1074/jbc.M110.130138 21212281 PMC3048689

[32] Whitnack E Beachey EH. Inhibition of complement-mediated opsonization and phagocytosis of *Streptococcus pyogenes* by D fragments of fibrinogen and fibrin bound to cell surface M protein. J Exp Med (1985) 162(6):1983–97. 10.1084/jem.162.6.1983 3906018 PMC2187975

[33] Carlsson F Sandin C Lindahl G. Human fibrinogen bound to *Streptococcus pyogenes* M protein inhibits complement deposition via the classical pathway. Mol Microbiol (2005) 56(1):28–39. 10.1111/j.1365-2958.2005.04527.x 15773976

[34] Hammerschmidt S Agarwal V Kunert A Haelbich S Skerka C Zipfel PF. The host immune regulator factor H interacts via two contact sites with the PspC protein of *Streptococcus pneumoniae* and mediates adhesion to host epithelial cells. J Immunol (2007) 178(9):5848–58. 10.4049/jimmunol.178.9.5848 17442969

[35] Ermert D Weckel A Agarwal V Frick IM Bjorck L Blom AM. Binding of complement inhibitor C4b-binding protein to a highly virulent *Streptococcus pyogenes* M1 strain is mediated by protein H and enhances adhesion to and invasion of endothelial cells. J Biol Chem (2013) 288(45):32172–83. 10.1074/jbc.M113.502955 24064215 PMC3820857

[36] Ram S McQuillen DP Gulati S Elkins C Pangburn MK Rice PA. Binding of complement factor H to loop 5 of porin protein 1A: a molecular mechanism of serum resistance of nonsialylated *Neisseria gonorrhoeae*. J Exp Med (1998) 188(4):671–80. 10.1084/jem.188.4.671 9705949 PMC2213355

[37] Hammerschmidt C Klevenhaus Y Koenigs A Hallstrom T Fingerle V Skerka C BGA66 and BGA71 facilitate complement resistance of *Borrelia bavariensis* by inhibiting assembly of the membrane attack complex. Mol Microbiol (2016) 99(2):407–24. 10.1111/mmi.13239 26434356

[38] Lahteenmaki K Kuusela P Korhonen TK. Plasminogen activation in degradation and penetration of extracellular matrices and basement membranes by invasive bacteria. Methods (2000) 21(2):125–32. 10.1006/meth.2000.0983 10816373

[39] Sun H. The interaction between pathogens and the host coagulation system. Physiology (Bethesda) (2006) 21:281–8. 16868317 10.1152/physiol.00059.2005

[40] Law RH Caradoc-Davies T Cowieson N Horvath AJ Quek AJ Encarnacao JA The X-ray crystal structure of full-length human plasminogen. Cell Rep (2012) 1(3):185–90. 10.1016/j.celrep.2012.02.012 22832192

[41] Kennedy AT Schmidt CQ Thompson JK Weiss GE Taechalertpaisarn T Gilson PR Recruitment of factor H as a novel complement evasion strategy for blood-stage *Plasmodium falciparum* infection. J Immunol (2016) 196(3):1239–48. 10.4049/jimmunol.1501581 26700768

[42] Collen D Lijnen HR Staphylokinase, a fibrin-specific plasminogen activator with therapeutic potential? Blood (1994) 84(3):680–6. 7519069

[43] Barthel D Schindler S Zipfel PF. Plasminogen is a complement inhibitor. J Biol Chem (2012) 287(22):18831–42. 10.1074/jbc.M111.323287 22451663 PMC3365705

[44] Attali C Frolet C Durmort C Offant J Vernet T Di Guilmi AM. *Streptococcus pneumoniae* choline-binding protein E interaction with plasminogen/plasmin stimulates migration across the extracellular matrix. Infect Immun (2008) 76(2):466–76. 10.1128/IAI.01261-07 18070889 PMC2223458

[45] Ghosh AK Coppens I Gardsvoll H Ploug M Jacobs-Lorena M. *Plasmodium* ookinetes coopt mammalian plasminogen to invade the mosquito midgut. Proc Natl Acad Sci U S A (2011) 108(41):17153–8. 10.1073/pnas.1103657108 21949403 PMC3193258

[46] Hallstrom T Morgelin M Barthel D Raguse M Kunert A Hoffmann R Dihydrolipoamide dehydrogenase of *Pseudomonas aeruginosa* is a surface-exposed immune evasion protein that binds three members of the factor H family and plasminogen. J Immunol (2012) 189(10):4939–50. 10.4049/jimmunol.1200386 23071278

[47] Funk J Schaarschmidt B Slesiona S Hallstrom T Horn U Brock M. The glycolytic enzyme enolase represents a plasminogen-binding protein on the surface of a wide variety of medically important fungal species. Int J Med Microbiol (2016) 306(1):59–68. 10.1016/j.ijmm.2015.11.005 26679571

[48] Banerjee B Greenberger PA Fink JN Kurup VP. Immunological characterization of Asp f 2, a major allergen from *Aspergillus fumigatus* associated with allergic bronchopulmonary aspergillosis. Infect Immun (1998) 66(11):5175–82. 9784519 10.1128/iai.66.11.5175-5182.1998 PMC108645

[49] Amich J Vicentefranqueira R Leal F Calera JA. *Aspergillus fumigatus* survival in alkaline and extreme zinc-limiting environments relies on the induction of a zinc homeostasis system encoded by the zrfC and aspf2 genes. Eukaryot Cell (2010) 9(3):424–37. 10.1128/EC.00348-09 20038606 PMC2837988

[50] da Silva Ferreira ME Kress MR Savoldi M Goldman MH Hartl A Heinekamp T The akuB(KU80) mutant deficient for nonhomologous end joining is a powerful tool for analyzing pathogenicity in *Aspergillus fumigatus*. Eukaryot Cell (2006) 5(1):207–11. 10.1128/EC.5.1.207-211.2006 16400184 PMC1360264

[51] Weidner G d’Enfert C Koch A Mol PC Brakhage AA. Development of a homologous transformation system for the human pathogenic fungus *Aspergillus fumigatus* based on the pyrG gene encoding orotidine 5’-monophosphate decarboxylase. Curr Genet (1998) 33(5):378–85. 10.1007/s002940050350 9618589

[52] Bergfeld A Dasari P Werner S Hughes TR Song WC Hortschansky P Direct binding of the pH-regulated protein 1 (Pra1) from *Candida albicans* inhibits cytokine secretion by mouse CD4+ T cells. Front Microbiol (2017) 8:844. 10.3389/fmicb.2017.00844 28553273 PMC5425473

[53] Poltermann S Kunert A von der Heide M Eck R Hartmann A Zipfel PF. Gpm1p is a factor H-, FHL-1-, and plasminogen-binding surface protein of *Candida albicans*. J Biol Chem (2007) 282(52):37537–44. 10.1074/jbc.M707280200 17959597

[54] Buhlmann D Eberhardt HU Medyukhina A Prodinger WM Figge MT Zipfel PF FHR3 blocks C3d-mediated coactivation of human B cells. J Immunol (2016) 197(2):620–9. 10.4049/jimmunol.1600053 27279373

[55] Szewczyk E Krappmann S. Conserved regulators of mating are essential for *Aspergillus fumigatus* cleistothecium formation. Eukaryot Cell (2010) 9(5):774–83. 10.1128/EC.00375-09 20348388 PMC2863953

[56] Valiante V Heinekamp T Jain R Hartl A Brakhage AA. The mitogen-activated protein kinase MpkA of *Aspergillus fumigatus* regulates cell wall signaling and oxidative stress response. Fungal Genet Biol (2008) 45(5):618–27. 10.1016/j.fgb.2007.09.006 17981060

[57] Dasari P Reiss K Lingelbach K Baumeister S Lucius R Udomsangpetch R Digestive vacuoles of *Plasmodium falciparum* are selectively phagocytosed by and impair killing function of polymorphonuclear leukocytes. Blood (2011) 118(18):4946–56. 10.1182/blood-2011-05-353920 21911835

[58] Meinel C Sparta G Dahse HM Horhold F Konig R Westermann M *Streptococcus pneumoniae* from patients with hemolytic uremic syndrome binds human plasminogen via the surface protein PspC and uses plasmin to damage human endothelial cells. J Infect Dis (2017) 217(3):358–70. 10.1093/infdis/jix305 28968817

[59] Bradski G The OpenCV Library Dr. Dobb’s Journal of Software Tools (2000). Available from: https://opencv.org/ (Accessed: July 10, 2018).

[60] Eric J Travis O Pearu P SciPy: Open Source Scientific Tools for Python (2001). Available from: http://www.scipy.org/ (Accessed: July 10, 2018).

[61] Brandes S Mokhtari Z Essig F Hunniger K Kurzai O Figge MT. Automated segmentation and tracking of non-rigid objects in time-lapse microscopy videos of polymorphonuclear neutrophils. Med Image Anal (2015) 20(1):34–51. 10.1016/j.media.2014.10.002 25465844

[62] Brandes S Dietrich S Hunniger K Kurzai O Figge MT. Migration and interaction tracking for quantitative analysis of phagocyte-pathogen confrontation assays. Med Image Anal (2017) 36:172–83. 10.1016/j.media.2016.11.007 27940225

[63] Citiulo F Jacobsen ID Miramon P Schild L Brunke S Zipfel P *Candida albicans* scavenges host zinc via Pra1 during endothelial invasion. PLoS Pathog (2012) 8(6):e1002777. 10.1371/journal.ppat.1002777 22761575 PMC3386192

[64] Luo S Dasari P Reiher N Hartmann A Jacksch S Wende E The secreted *Candida albicans* protein Pra1 disrupts host defense by broadly targeting and blocking complement C3 and C3 activation fragments. Mol Immunol (2017) 93:266–77. 10.1016/j.molimm.2017.07.010 28860090

[65] Haupt K Reuter M van den Elsen J Burman J Halbich S Richter J The *Staphylococcus aureus* protein Sbi acts as a complement inhibitor and forms a tripartite complex with host complement Factor H and C3b. PLoS Pathog (2008) 4(12):e1000250. 10.1371/journal.ppat.1000250 19112495 PMC2602735

[66] Chen J Ryu S Gharib SA Goodlett DR Schnapp LM. Exploration of the normal human bronchoalveolar lavage fluid proteome. Proteomics Clin Appl (2008) 2(4):585–95. 10.1002/prca.200780006 21136857 PMC4432467

[67] Bartlett JA Albertolle ME Wohlford-Lenane C Pezzulo AA Zabner J Niles RK Protein composition of bronchoalveolar lavage fluid and airway surface liquid from newborn pigs. Am J Physiol Lung Cell Mol Physiol (2013) 305(3):L256–66. 10.1152/ajplung.00056.2013 23709621 PMC3743012

[68] Caesar JJ Lavender H Ward PN Exley RM Eaton J Chittock E Competition between antagonistic complement factors for a single protein on *N. meningitidis* rules disease susceptibility. Elife (2014) 3. 10.7554/eLife.04008 25534642 PMC4273445

[69] Meri T Amdahl H Lehtinen MJ Hyvarinen S McDowell JV Bhattacharjee A Microbes bind complement inhibitor factor H via a common site. PLoS Pathog (2013) 9(4):e1003308. 10.1371/journal.ppat.1003308 23637600 PMC3630169

[70] Knutsen AP Hutcheson PS Slavin RG Kurup VP. IgE antibody to *Aspergillus fumigatus* recombinant allergens in cystic fibrosis patients with allergic bronchopulmonary aspergillosis. Allergy (2004) 59(2):198–203. 10.1046/j.1398-9995.2003.00310.x 14763934

[71] Zipfel PF Skerka C Kupka D Luo S. Immune escape of the human facultative pathogenic yeast *Candida albicans*: the many faces of the *Candida* Pra1 protein. Int J Med Microbiol (2011) 301(5):423–30. 10.1016/j.ijmm.2011.04.010 21565550

