# Supplementary material for: Aspf2 From Aspergillus fumigatus Recruits Human Immune Regulators for Immune Evasion and Cell Damage
Source: Front Immunol. 2018 Aug 3;9:1635. doi: 10.3389/fimmu.2018.01635 (PMC6106110; doi:10.3389/fimmu.2018.01635)
Supplement: Supplementary file 1 [file Presentation_1.PDF]

Supplementary Information

Aspf2 from *Aspergillus fumigatus* recruits human immune regulators for immune evasion and cell damage

Prasad Dasari[1], Iordana A. Shopova[2], Maria Stroe[2], Dirk Wartenberg[2], Hans Martin-Dahse[1], Niklas Beyersdorf[3], Peter Hortschansky[2], Stefanie Dietrich[4,5], Zoltán Cseresnyés[4], Marc Thilo Figge[4,5], Martin Westermann[6], Christine Skerka[1], Axel A. Brakhage[2,5], Peter F. Zipfel[1,5,*]

[1]Department of Infection Biology, Leibniz Institute for Natural Product Research and Infection Biology, Hans Knöll Institute (HKI), Jena, Germany;

[2]Department of Molecular and Applied Microbiology, Leibniz Institute for Natural Product Research and Infection Biology (HKI), Jena, Germany;

[3]University of Würzburg, Institute for Virology and Immunobiology, Würzburg, Germany;

[4]Research Group Applied Systems Biology, Leibniz Institute for Natural Product Research and Infection Biology (HKI), Jena, Germany;

[5]Faculty for Biological Sciences, Friedrich Schiller University, Jena, Germany.

[6]Electron Microscopy Center of the University Hospital, Jena, Germany;

*Address correspondence to Peter F. Zipfel, Department of Infection Biology, Leibniz Institute for Natural Product Research and Infection Biology (HKI), Beutenbergstr. 11a, 07745 Jena, Germany. Phone: +49 (0)3641 532-1300; Fax: +49 (0)3641 532-0807; E-mail: peter.zipfel@leibniz-hki.de

Supplementary Figure 1

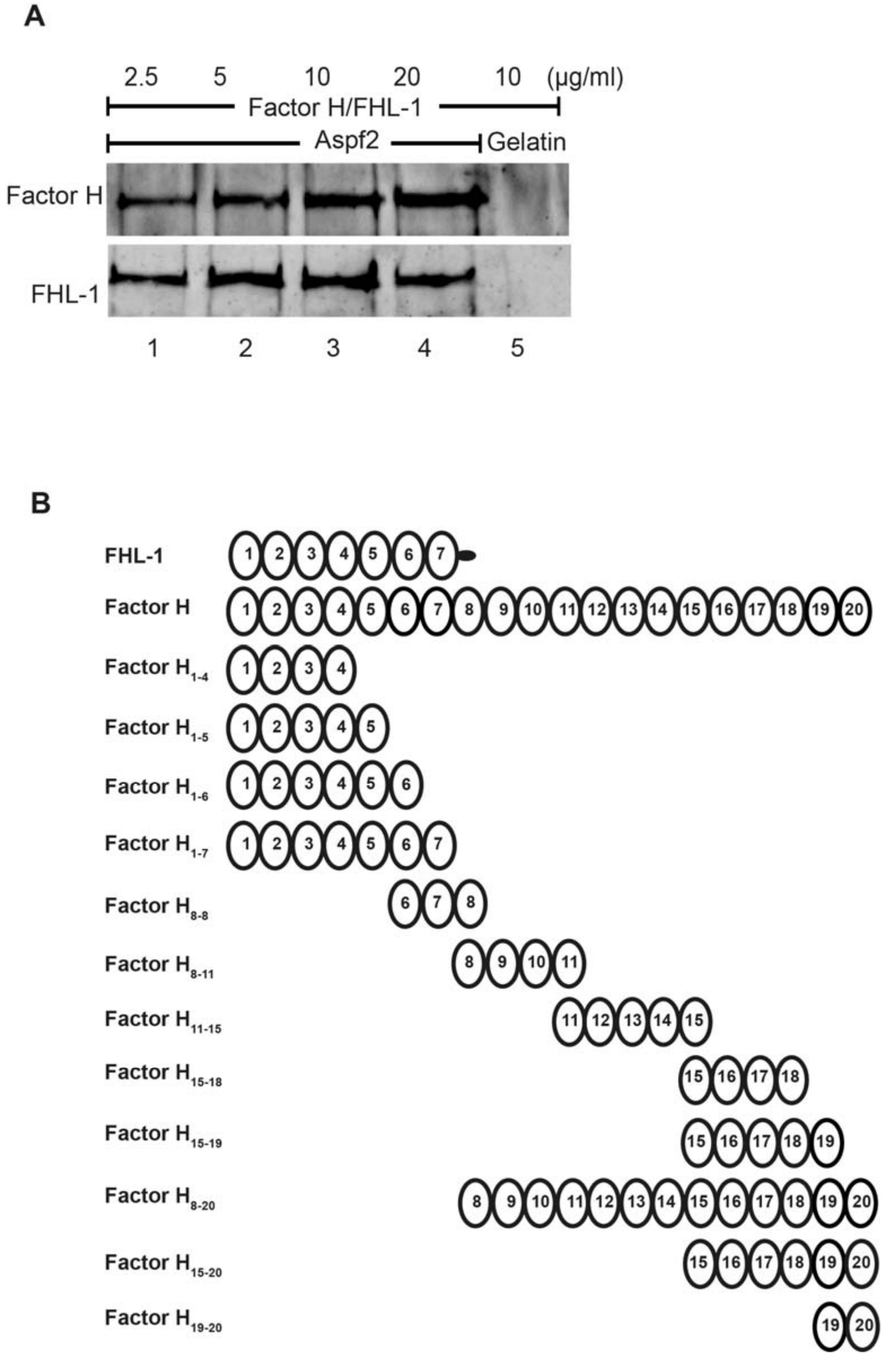

Supplementary Figure 1: Factor H and FHL-1 to immobilized Aspf2. (**A**) Factor H (upper panel) or FHL-1 (lower panel) used at the indicated amounts was bound to immobilized Aspf2. After washing, bound proteins were eluted, separated by SDS-PAGE, and transferred to a membrane. Factor H (or FHL-1) was identified by with goat anti-human Factor H antiserum. Factor H as well as FHL1 bound to Aspf2 dose-dependently (lanes 1-5). Neither Factor H nor FHL-1 bound to gelatin (lane 6). (**B**) Diagram showing domain structure of Factor H and FHL1 and the structure of the **Factor H/FHL-1** deletion mutants used. The names identify the SCR-domains that are contained in the mutants.

Supplementary Figure 2

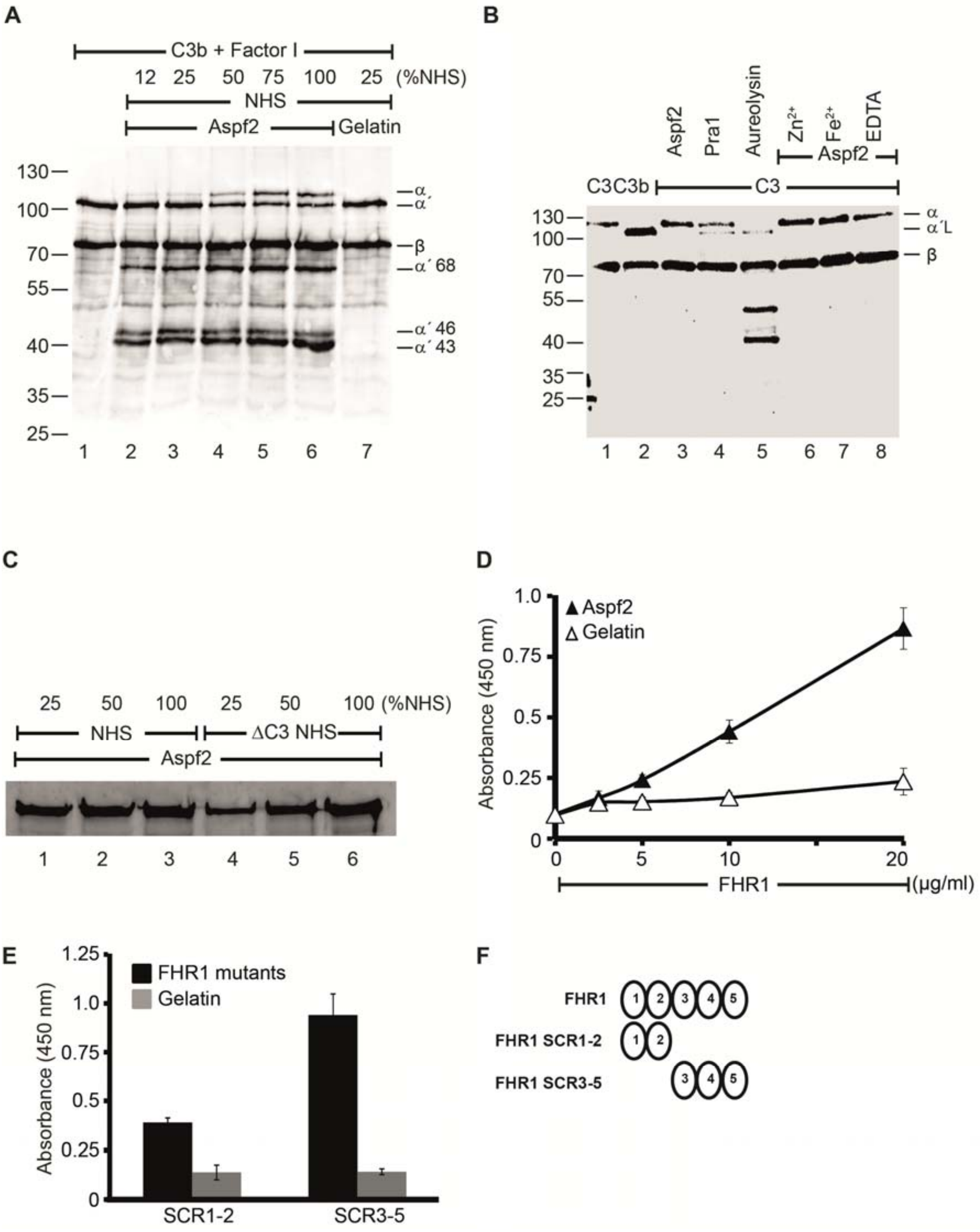

Supplementary Figure 2: (**A**) **Factor H and FHL-1 from NHS bound to Aspf2 retain cofactor activity.** NHS-EDTA (10 mM EDTA) at the indicated concentrations was added to immobilized Aspf2. After washing, C3b and Factor I were added. Following 1 h incubation, the proteins were separated by SDS-PAGE and transferred to a membrane. C3b cleavage products were identified by Western blotting. Factor H/FHL1 attached to Aspf2 maintained cofactor activity for Factor I as revealed by the increased intensity of the α–68', α-46' and α-43' kDa bands (lane 2-6). No cleavage occurred when Factor H was added to the gelatin (lane 7). An additional band of 115 kDa appeared which most likely represents the intact α band of C3. (**B**) **Aspf2 lacks C3 cleaving activity.** C3 was combined with Aspf2, Pra1 (*C. albicans)* or aureolysin (the C3 cleaving metalloprotease from *Staphylococcus aureus*). The mixtures were incubated for 1 h at 37 °C and then separated by SDS-PAGE. After transfer to a membrane C3 cleavage products were identified with goat anti-human C3 antiserum. C3 combined with Aspf2 (lane 3) remained intact and no cleavage bands were detected.

C3 combined either with Pra1 or aureolysin was cleaved (lanes 4 and 5) and new bands α'L and in case of aureolysin additional band of ca 52 and 43 kDa bands were identified. Neither metal ions, i.e. Zn^{2+}, Fe^{2+} (lanes 6 and 7) nor in presence of a metalloprotease inhibitor EDTA did induce a proteolytic activity of Aspf2. (**C**) **Factor H derived from NHS bound to Aspf2, and binding is also occurs in the absence of C3.** NHS was added to Aspf2**.** After washing, bound proteins were eluted, separated by SDS-PAGE, transferred to a membrane and Factor H was detected with goat anti-human Factor H antiserum. Factor derived from NHS (lanes 1-3), as well as from C3 depleted serum (ΔC3NHS) bound to Aspf2. Binding was dose-dependent (lanes 4-6). (**D**) **Aspf2 binding to FHR1 was studied by ELISA**. Aspf2 at the indicated amounts was added to immobilized FHR1, and bound Aspf2 was detected with **mAb anti-Aspf2 (1G12**). Aspf2 bound to FHR1, and binding was dose-dependent. (**E**) **Localization of Aspf2 binding domain in FHR1**. Aspf2 was added to the immobilized FHR1 deletion mutants (SCR1–2 and SCR3–5), and bound Aspf2 was analyzed. Aspf2 binds to the C-terminal domain of FHR1 i.e. SCR3–5. (**F**) Schematic representation of FHR1 deletion mutants and used to determine the binding site of Aspf2 to FHR1. Name indicates the domains they retain.

Supplementary Figure 3

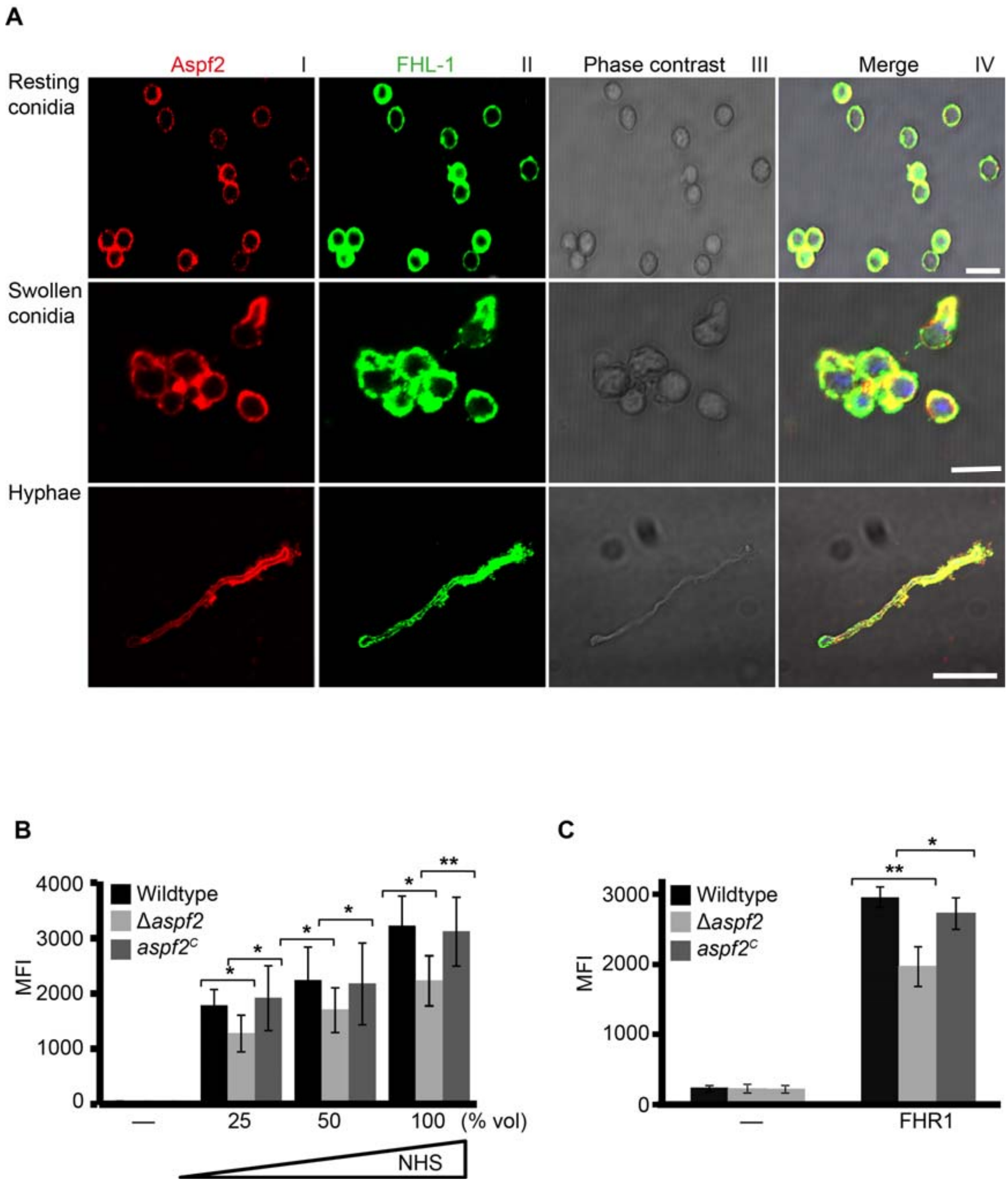

Supplementary Figure 3: FHL-1 and Aspf2 colocalize on the surface of *A. fumigatus*. (**A**) FHL-1 and Aspf2 co-localize on the surface *of A. fumigatus*. Purified FHL-1 was added to resting, swollen conidia, and hyphae. After washing, the cells were stained with goat anti-human Factor H antiserum, and mouse anti-Aspf2 antiserum, followed by corresponding secondary antibodies. FHL-1 and Aspf2 colocalized on the surface of resting-(upper panel), swollen conidia (middle panel), and hyphae (lower panel). (**B**) **Δ*aspf2* conidia recruit less Factor H from NHS.** Δ*aspf2*, wildtype, and *aspf2*^{C} conidia were incubated with NHS used at increasing concentrations, and bound Factor H was analyzed by flow cytometry. When NHS used at 100%, Δ*aspf2* conidia recruited 31% less Factor H than wildtype and *aspf2*^{C} conidia. (C) **The Δ*aspf2* conidia binds FHR1 with lower intensity**. FHR1 (10 µg/ml) was incubated with Δ*aspf2*, wildtype, and conidia, and surface bound FHR1 was determined by flow cytometry. FHR1 bound with 33% reduced intensity to the Δ*aspf2* strain.

Supplementary Figure 4

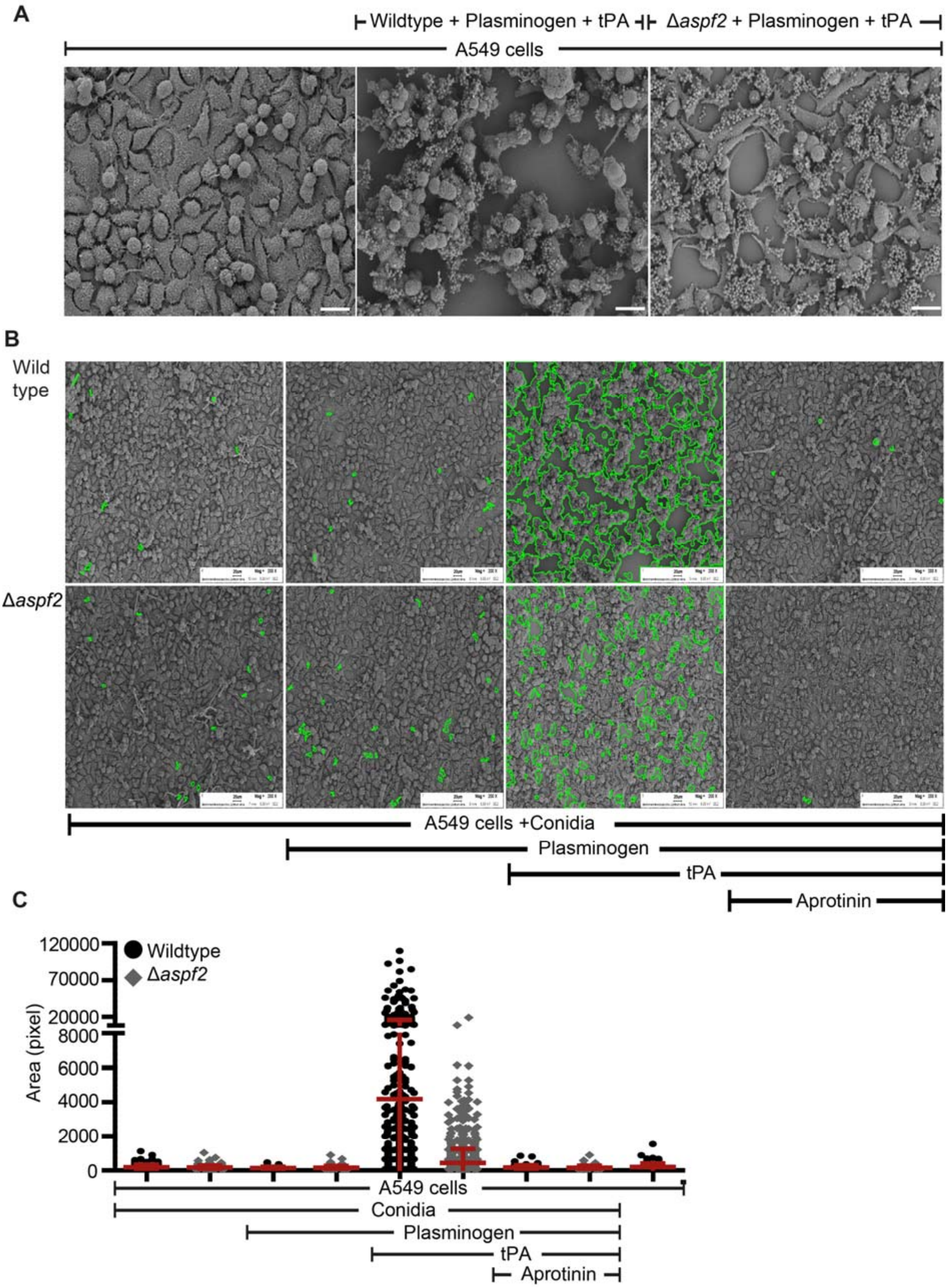

Supplementary Figure 4: Plasminogen bound to conidia is converted to plasmin which damages human lung epithelial cells and induces cell retraction. Bioinformatics image quantification of cell retraction. (A) **Morphological changes of lung epithelial cells upon challenge with plasmin-bound conidia were visualized by scanning electron microscopy.** Intact human lung epithelial cell monolayer (left panel), epithelial cells challenged with plasmin-coated wildtype conidia (middle panel) and epithelial cells challenged with plasmin coated with Δ*aspf2* conidia (left panel) are shown. Bars: 20 µm**.** (**B**) **Visualization of cell**

retraction by electron microscopy. Plasminogen was added to wildtype (upper panel) or Δ*aspf2* conidia (lower panel). After washing, the conidia were added to confluent human lung epithelial cells (A549) together with tPA. Following 2 h incubation, unbound conidia were washed off, and A549 cells were fixed with 2.5 % glutaraldehyde/cacodylate. For electron microscopy A549 cells were treated with 1 % osmium tetroxide/cacodylate and dehydrated. Multiple images were acquired with a Zeiss 1530 FESEM. Plasminogen bound to wildtype conidia when activated to plasmin induced cell retraction, resulting in exposure of underlying matrix (upper row, column III). Similarly plasmin bound to Δ*aspf2* conidia induced cell retraction. In this case cell retraction was not as pronounced (lower row, column III). The serine protease inhibitor aprotinin blocked plasmin activity (columns on the right). The automatically identified areas of exposed surface matrix are marked in the images above by green contours. (**C**) **Bioinformatics-based image quantification of cell retraction.** Using the electron microscopy images (10 images) generated in supplementary Figure 4B, the numbers of damaged spots formed and the void area per spot were quantified by a novel bioinformatics-based image analysis. Active plasmin on the surface of wildtype conidia induced larger damage than the Δ*aspf2* conidia. Aprotinin inhibited plasmin activity. The mean values are indicated by the horizontal red bar, and the SD is shown by the vertical red bars.
